# Neuroanatomy of the mekosuchine crocodylian *Trilophosuchus rackhami* Willis, 1993

**DOI:** 10.1111/joa.13732

**Published:** 2022-08-29

**Authors:** Jorgo Ristevski

**Affiliations:** ^1^ School of Biological Sciences The University of Queensland Brisbane Queensland Australia

**Keywords:** Australia, Crocodylia, Crocodyliformes, Crocodylomorpha, Mekosuchinae, paleoneurology, *Trilophosuchus*

## Abstract

Although our knowledge on crocodylomorph palaeoneurology has experienced considerable growth in recent years, the neuroanatomy of many crocodylomorph taxa has yet to be studied. This is true for Australian taxa, where thus far only two crocodylian crocodylomorphs have had aspects of their neuroanatomy explored. Here, the neuroanatomy of the Australian mekosuchine crocodylian *Trilophosuchus rackhami* is described for the first time, which significantly increases our understanding on the palaeoneurology of Australian crocodylians. The palaeoneurological description is based on the taxon's holotype specimen (QMF16856), which was subjected to a μCT scan. Because of the exceptional preservation of QMF16856, most neuroanatomical elements could be digitally reconstructed and described in detail. Therefore, the palaeoneurological assessment presented here is hitherto the most in‐depth study of this kind for an extinct Australian crocodylomorph. *Trilophosuchus rackhami* has a brain endocast with a distinctive morphology that is characterized by an acute dural peak over the hindbrain region. While the overall morphology of the brain endocast is unique to *T. rackhami*, it does share certain similarities with the notosuchian crocodyliforms *Araripesuchus wegeneri* and *Sebecus icaeorhinus*. The endosseous labyrinth displays a morphology that is typical for crocodylians, although a stand‐out feature is the unusually tall common crus. Indeed, the common crus of *T. rackhami* has one of the greatest height ratios among crocodylomorphs with currently known endosseous labyrinths. The paratympanic pneumatic system of *T. rackhami* is greatly developed and most similar to those of the extant crocodylians *Osteolaemus tetraspis* and *Paleosuchus palpebrosus*. The observations on the neuroanatomy of *T. rackhami* are also discussed in the context of Crocodylomorpha. The comparative palaeoneurology reinforces previous evaluations that the neuroanatomy of crocodylomorphs is complex and diverse among species, and *T. rackhami* has a peculiar neuromorphology, particularly among eusuchian crocodyliforms.

## Graphical abstract

This study described the neuroanatomy of the mekosuchine crocodylian *Trilophosuchus rackhami* from the Middle Miocene of Australia. Although the neuromorphology of *Trilophosuchus rackhami* follows the general patterns seen in crocodylomorphs, it is nevertheless characterized by a unique combination of features that stand out among currently known taxa. The brain endocast has a peculiar acute dural peak over the hindbrain region. The endosseous labyrinth has an unusually tall common crus, with a heigh ratio greater than any other eusuchian for which there is palaeoneurological data. The braincase of *Trilophosuchus rackhami* is greatly pneumatized.

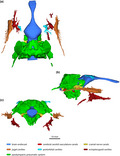

## 1 INTRODUCTION

Today, Australia is inhabited by two crocodylian species, both of which belong to the genus *Crocodylus* Laurenti, 1768. These are *Crocodylus johnstoni* (Krefft, 1873) and *Crocodylus porosus* Schneider, 1801, with *C. johnstoni* being endemic to mainland northern Australia (Isberg et al., 2017), whereas *C. porosus* inhabits northern Australia, India, New Guinea, Vanuatu, as well as a vast range in southeast Asia (Webb et al., 2021). In contrast, Australia's Cenozoic fossil record is represented by a remarkably rich crocodylian fauna, with crocodylian remains being known from multiple localities across the continent (Willis, 2006). The oldest known crocodylian fossils from the Cenozoic of Australia date back to the Eocene (Buchanan, 2009; Holt et al., 2005; Salisbury & Willis, 1996; Willis et al., 1993; see also Willis & Molnar, 1991) whereas the youngest are from the Pleistocene (Molnar, 1981; Ristevski et al., 2020a; Willis & Archer, 1990; Willis & Molnar, 1997). As currently understood, the majority of crocodylian taxa from the Cenozoic Era of Australia belong to the now extinct clade Mekosuchinae. Traditionally, Mekosuchinae has been considered a subfamily of Crocodylidae (Brochu, 2001; Willis, 1997b), although recent phylogenetic studies have recovered Mekosuchinae outside of Crocodylidae (Azzarà et al., 2021; Cossette et al., 2020; Lee & Yates, 2018; Rio & Mannion, 2021; Ristevski et al., 2022). Other than Mekosuchinae and *Crocodylus*, Australia was also inhabited by members of Gavialoidea—*Gunggamarandu maunala* Ristevski et al., 2021 from the Pliocene or Pleistocene of Queensland, and *Harpacochampsa camfieldensis* Megirian et al., 1991 from the Middle Miocene of the Northern Territory. Extinct crocodylians from Australia's Cenozoic display substantial morphological disparities between them, including small‐bodied (~2 m in TL or less) to large (over four meters in TL) taxa, as well as differences in snout (e.g., broad‐snouted or brevirostrine, long and slender‐snouted, and tall‐snouted or altirostral) and dental (e.g., conidont or conical and non‐serrated teeth, as well as ziphodont or labiolingually compressed and serrated teeth) morphologies that indicate different feeding adaptations and palaeoecologies (Willis, 2006).

**FIGURE 1:**
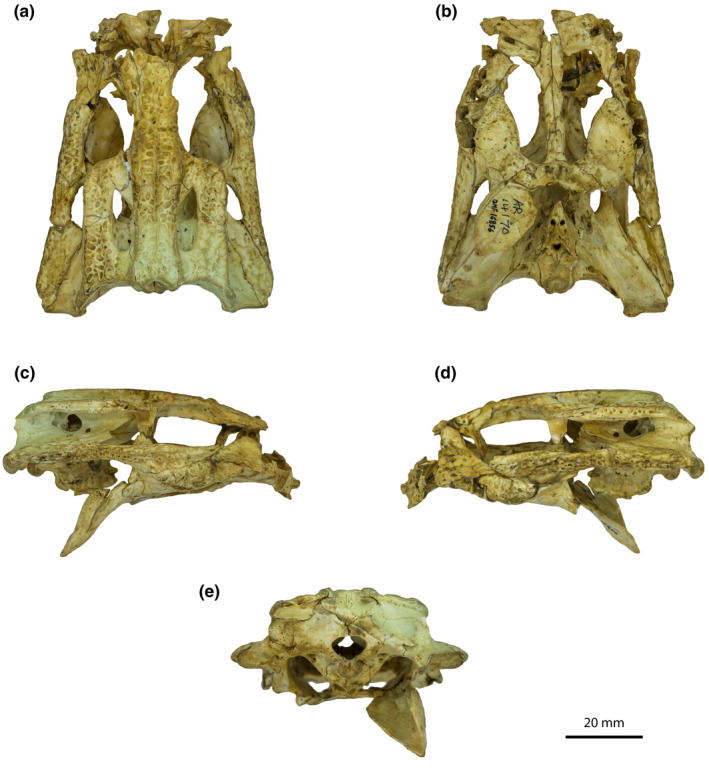
*Trilophosuchus rackhami* Willis, 1993, QMF16856, holotype. Cranium in (a) dorsal, (b) ventral, (c) right lateral, (d) left lateral, and (e) posterior views.

One of the most bountiful fossil localities from Australia is the Riversleigh World Heritage Area (WHA) in northwestern Queensland (Archer et al., 2000, 2006). Although best known for its palaeodiversity of Middle–Late Cenozoic mammals (Archer et al., 2006) the Riversleigh WHA has also yielded numerous crocodylian remains (e.g., Stein et al., 2016; Willis, 1993, 1997a, 2001; Willis & Archer, 1990; Willis & Molnar, 1997), including those of *Trilophosuchus rackhami* Willis, 1993. The mekosuchine genus *Trilophosuchus* Willis, 1993 contains one named species that is known from only three specimens. *Trilophosuchus rackhami* is best represented by its holotype, QMF16856 (formerly AR 14170), an incomplete skull with an excellently preserved braincase (Figure 1). *Trilophosuchus rackhami* was a small‐bodied crocodylian with a short altirostral snout and a unique cranial table morphology characterized by three continuous longitudinal crests (Ristevski et al., 2022; Willis, 1993). The phylogenetic relationships of *T. rackhami* have been tested in many cladistic analyses, and all recovered it within Mekosuchinae (e.g., Azzarà et al., 2021; Brochu, 2001, 2003, 2007, 2012; Cossette et al., 2020; Lee & Yates, 2018; Molnar et al., 2002; Rio & Mannion, 2021; Ristevski et al., 2022; Salisbury & Willis, 1996; Scheyer et al., 2013; Stein et al., 2016; Willis, 1993, 1995; Yates & Pledge, 2016).

The holotype of *T. rackhami* was recovered at the Ringtail Site of the Riversleigh WHA, northern Gag Plateau, Riversleigh Station. Two additional specimens from Ringtail Site, QMF16857 (an isolated frontal) and QMF16858 (an isolated right postorbital), are assigned to *T. rackhami* (Ristevski et al., 2022). Therefore, the species *T. rackhami* is presently confirmed only from this locality. Radiometric dating of the Ringtail Site points to a Middle Miocene age (maximum estimate of 14.2 Ma, late Langhian, and minimum estimate of 12.9 Ma, middle Serravallian; Woodhead et al., 2016). In addition to *T. rackhami*, the Ringtail Site has produced fossils of other mekosuchines such as *Mekosuchus sanderi* Willis, 2001 as well as remains referrable to *Baru* Willis et al., 1990 (Archer et al., 2006; Willis, 2001; Yates, 2017). An isolated parietal (QMF60374) from the Hiatus Site at the Riversleigh WHA is also referrable to the genus *Trilophosuchus* (Ristevski et al., 2022). The age of the Hiatus Site is estimated as late Oligocene based on topographic and faunal relationships (Archer et al., 1997; Scanlon, 2006a, 2006b), though radiometric dates have not been provided (Woodhead et al., 2016). While the specific assignment of QMF60374 is currently undetermined (referred to as *Trilophosuchus* sp.), it demonstrates that the fossil record of the genus *Trilophosuchus* goes back to the late Oligocene (~25 Ma). At present, no mandibular, dental, or postcranial elements can be assigned to *Trilophosuchus* with confidence.

**FIGURE 2:**
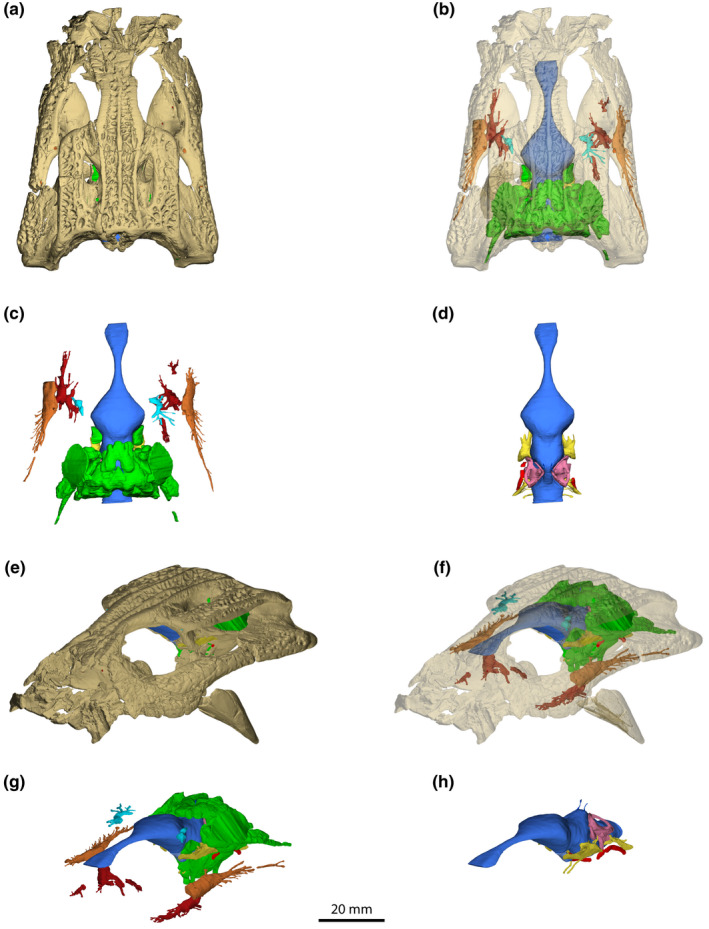
*Trilophosuchus rackhami* Willis, 1993, QMF16856, holotype. (a) Opaque digital model of the cranium in dorsal view. (b) Transparent digital model of the cranium in dorsal view, exposing the endocranial elements. (c) Endocranial elements in dorsal view. (d) Endocranial elements, excluding paratympanic pneumatic system and some vascular and neurovascular cavities, in dorsal view. (e) Opaque digital model of the cranium in oblique anterolateral view. (f) Transparent digital model of the cranium in oblique anterolateral view. (g) Endocranial elements in oblique anterolateral view. (h) Endocranial elements, excluding paratympanic pneumatic system and some vascular and neurovascular cavities, in oblique anterolateral view.

**FIGURE 3:**
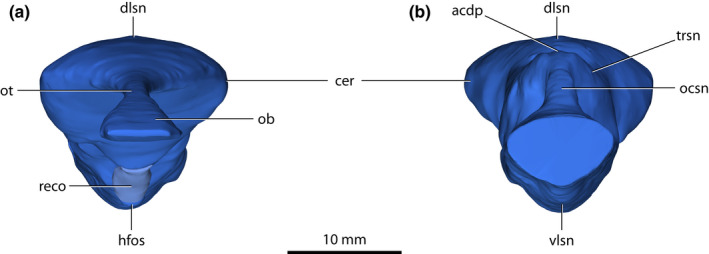
*Trilophosuchus rackhami* Willis, 1993, QMF16856, holotype. Brain endocast in (a) anterior, and (b) posterior views. Note that the endocast of the olfactory bulbs could not be reconstructed in its entirety. Abbreviations: acdp, acute dorsal dural peak; cer, cerebrum (endocast); dlsn, dorsal longitudinal dural venous sinus (endocast); hfso, hypophyseal fossa; ob, olfactory bulb (endocast); ocsn, occipital dural venous sinus (endocast); ot, olfactory tract (endocast); reco, digitally reconstructed missing portion of the hypophyseal fossa; trsn, transverse dural venous sinus (endocast); vlsn, ventral longitudinal dural venous sinus (endocast).

**FIGURE 4:**
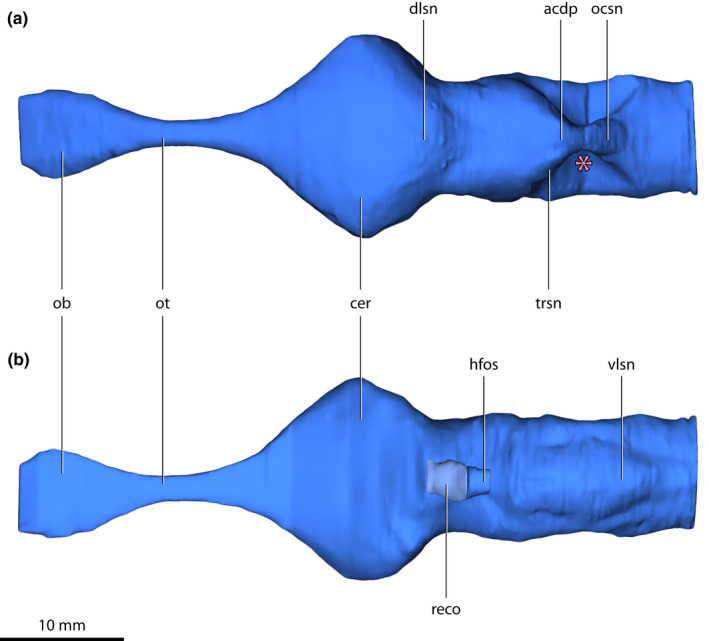
*Trilophosuchus rackhami* Willis, 1993, QMF16856, holotype. Brain endocast in (a) dorsal, and (b) ventral views. The pink asterisk marks the concave area on the endocast where the endosseous labyrinth is situated. Note that the endocast of the olfactory bulbs could not be reconstructed in its entirety. Abbreviations: acdp, acute dorsal dural peak; cer, cerebrum (endocast); dlsn, dorsal longitudinal dural venous sinus (endocast); hfso, hypophyseal fossa; ob, olfactory bulb (endocast); ocsn, occipital dural venous sinus (endocast); ot, olfactory tract (endocast); reco, digitally reconstructed missing portion of the hypophyseal fossa; trsn, transverse dural venous sinus (endocast); vlsn, ventral longitudinal dural venous sinus (endocast).

**FIGURE 5:**
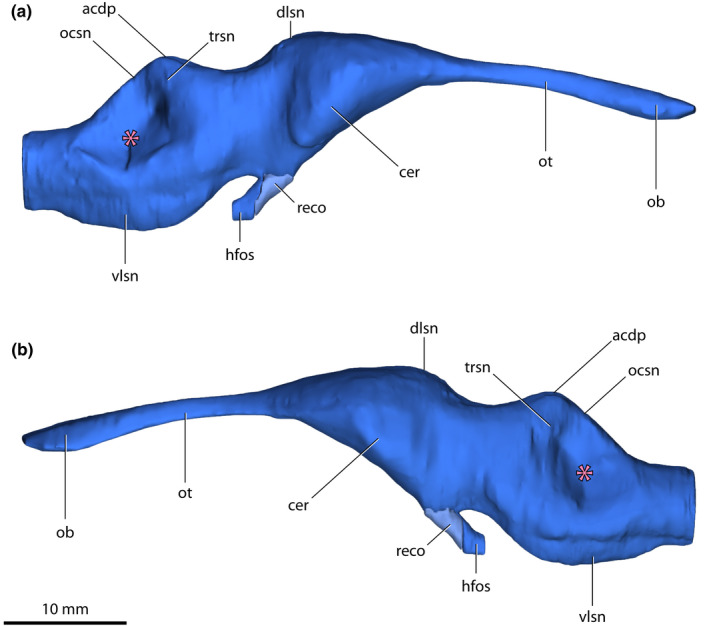
*Trilophosuchus rackhami* Willis, 1993, QMF16856, holotype. Brain endocast in (a) right lateral, and (b) left lateral views. The pink asterisk marks the concave area on the endocast where the endosseous labyrinth is situated. Note that the endocast of the olfactory bulbs could not be reconstructed in its entirety. Abbreviations: acdp, acute dorsal dural peak; cer, cerebrum (endocast); dlsn, dorsal longitudinal dural venous sinus (endocast); hfso, hypophyseal fossa; ob, olfactory bulb (endocast); ocsn, occipital dural venous sinus (endocast); ot, olfactory tract (endocast); reco, digitally reconstructed missing portion of the hypophyseal fossa; trsn, transverse dural venous sinus (endocast); vlsn, ventral longitudinal dural venous sinus (endocast).

**FIGURE 6:**
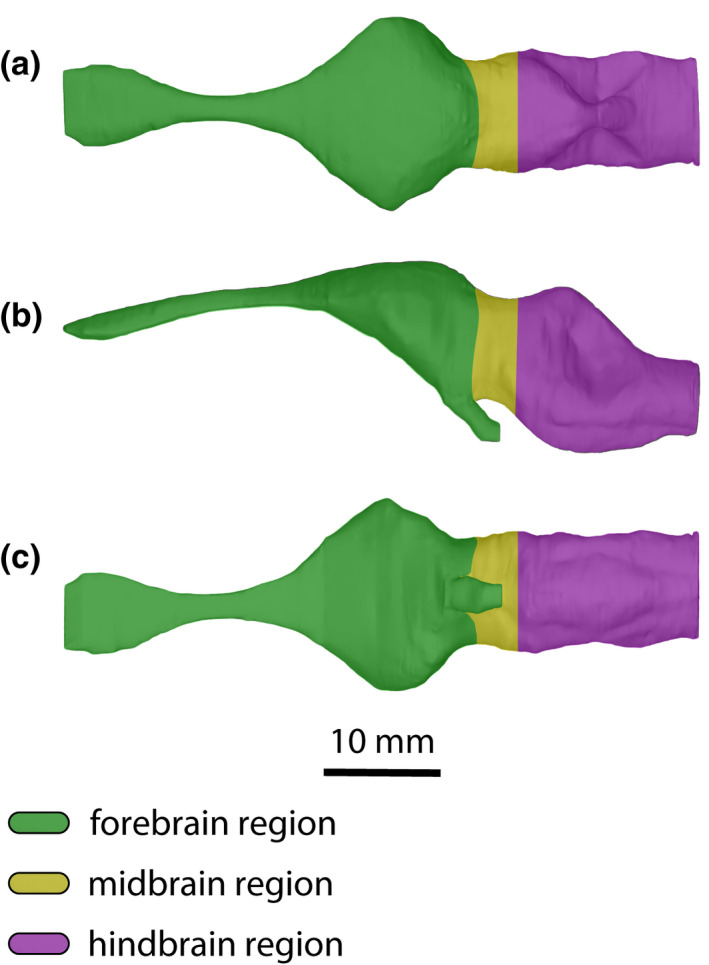
*Trilophosuchus rackhami* Willis, 1993, QMF16856, holotype. Brain endocast in (a) dorsal, (b) left lateral, and (c) ventral views. The regions on the endocast corresponding with the three major brain divisions are highlighted with different colors.

**FIGURE 7:**
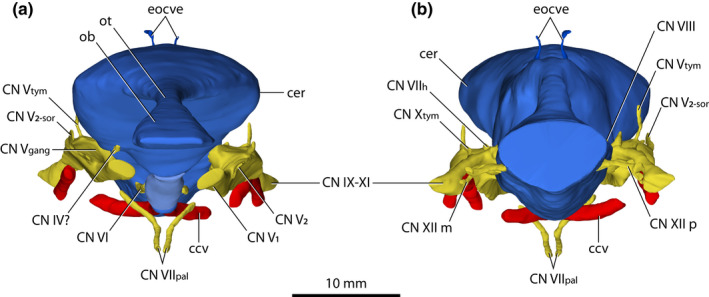
*Trilophosuchus rackhami* Willis, 1993, QMF16856, holotype. Brain endocast, cranial nerve canals, external occipital vein canals, and cerebral carotid vasculature canals in (a) anterior, and (b) posterior views. Emphasis is given on the cranial nerve canals and cerebral carotid vasculature canals. The endosseous labyrinths are intentionally not shown in order to better expose certain cranial nerve canals. Note that the cerebral carotid vasculature canals could not be reconstructed in their entirety. Abbreviations: ccv, cerebral carotid vasculature canal; cer, cerebrum (endocast); CN IV?, trochlear nerve canal (tentative); CN IX–XI, shared canal for the glossopharyngeal, vagus, and accessory nerves and accompanying vessels; CN V_1_, ophthalmic nerve canal; CN V_2_, maxillary nerve canal; CN V_2‐sor_, canal for the supraorbital branch of the maxillary nerve; CN V_gang_, trigeminal (Gasserian) ganglion (endocast); CN V_tym_, canal for the tympanic branch of the trigeminal nerve; CN VI, abducens canal; CN VII_h_, canal for the hyomandibular branch of facial nerve; CN VII_pal_, canal for the palatine branch of facial nerve; CN VIII, canals for vestibulocochlear nerve; CN X_tym_, canal for the tympanic branch of glossopharyngeal and vagus nerves; CN XII m, middle hypoglossal canal; CN XII p, posterior hypoglossal canal; eocve, external occipital vein canals; ob, olfactory bulb (endocast); ot, olfactory tract (endocast).

**FIGURE 8:**
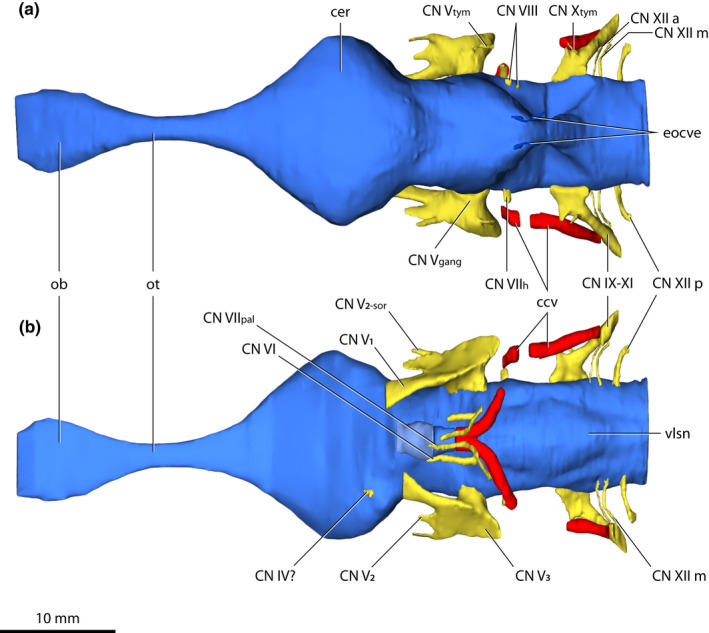
*Trilophosuchus rackhami* Willis, 1993, QMF16856, holotype. Brain endocast, cranial nerve canals, external occipital vein canals, and cerebral carotid vasculature canals in (a) dorsal, and (b) ventral views. Emphasis is given on the cranial nerve canals and cerebral carotid vasculature canals. The endosseous labyrinths are intentionally not shown in order to better expose certain cranial nerve canals. Note that the cerebral carotid vasculature canals could not be reconstructed in their entirety. Abbreviations: ccv, cerebral carotid vasculature canal; cer, cerebrum (endocast); CN IV?, trochlear nerve canal (tentative); CN IX–XI, shared canal for the glossopharyngeal, vagus, and accessory nerves and accompanying vessels; CN V_1_, ophthalmic nerve canal; CN V_2_, maxillary nerve canal; CN V_2‐sor_, canal for the supraorbital branch of the maxillary nerve; CN V_3_, mandibular nerve canal; CN V_gang_, trigeminal (Gasserian) ganglion (endocast); CN V_tym_, canal for the tympanic branch of the trigeminal nerve; CN VI, abducens canal; CN VII_h_, canal for the hyomandibular branch of facial nerve; CN VII_pal_, canal for the palatine branch of facial nerve; CN VIII, canals for vestibulocochlear nerve; CN X_tym_, canal for the tympanic branch of glossopharyngeal and vagus nerves; CN XII a, anterior hypoglossal canal; CN XII m, middle hypoglossal canal; CN XII p, posterior hypoglossal canal; eocve, external occipital vein canals; ob, olfactory bulb (endocast); ot, olfactory tract (endocast); vlsn, ventral longitudinal dural venous sinus (endocast).

**FIGURE 9:**
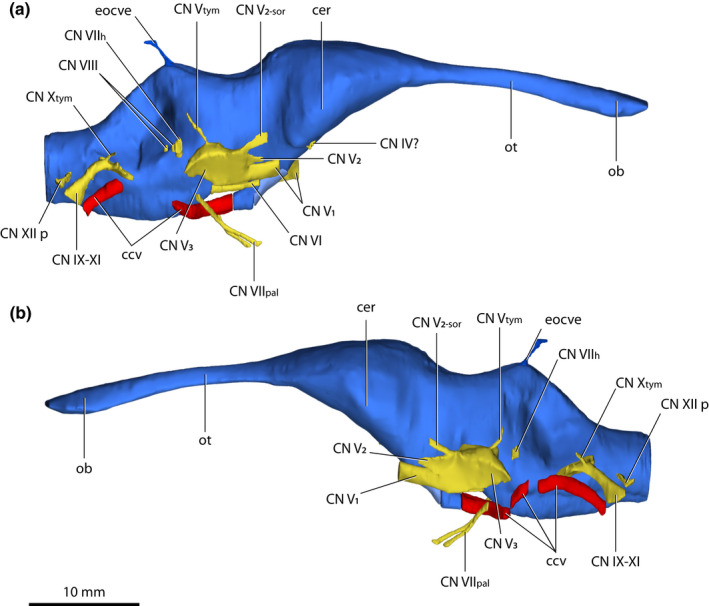
*Trilophosuchus rackhami* Willis, 1993, QMF16856, holotype. Brain endocast, cranial nerve canals, external occipital vein canals, and cerebral carotid vasculature canals in (a) right lateral, and (b) left lateral views. Emphasis is given on the cranial nerve canals and cerebral carotid vasculature canals. The endosseous labyrinths are intentionally not shown in order to better expose certain cranial nerve canals. Note that the cerebral carotid vasculature canals could not be reconstructed in their entirety. Abbreviations: ccv, cerebral carotid vasculature canal; cer, cerebrum (endocast); CN IV?, trochlear nerve canal (tentative); CN IX–XI, shared canal for the glossopharyngeal, vagus, and accessory nerves and accompanying vessels; CN V_1_, ophthalmic nerve canal; CN V_2_, maxillary nerve canal; CN V_2‐sor_, canal for the supraorbital branch of the maxillary nerve; CN V_3_, mandibular nerve canal; CN V_tym_, canal for the tympanic branch of the trigeminal nerve; CN VI, abducens canal; CN VII_h_, canal for the hyomandibular branch of facial nerve; CN VII_pal_, canal for the palatine branch of facial nerve; CN VIII, canals for vestibulocochlear nerve; CN X_tym_, canal for the tympanic branch of glossopharyngeal and vagus nerves; CN XII p, posterior hypoglossal canal; eocve, external occipital vein canal; ob, olfactory bulb (endocast); ot, olfactory tract (endocast).

**FIGURE 10:**
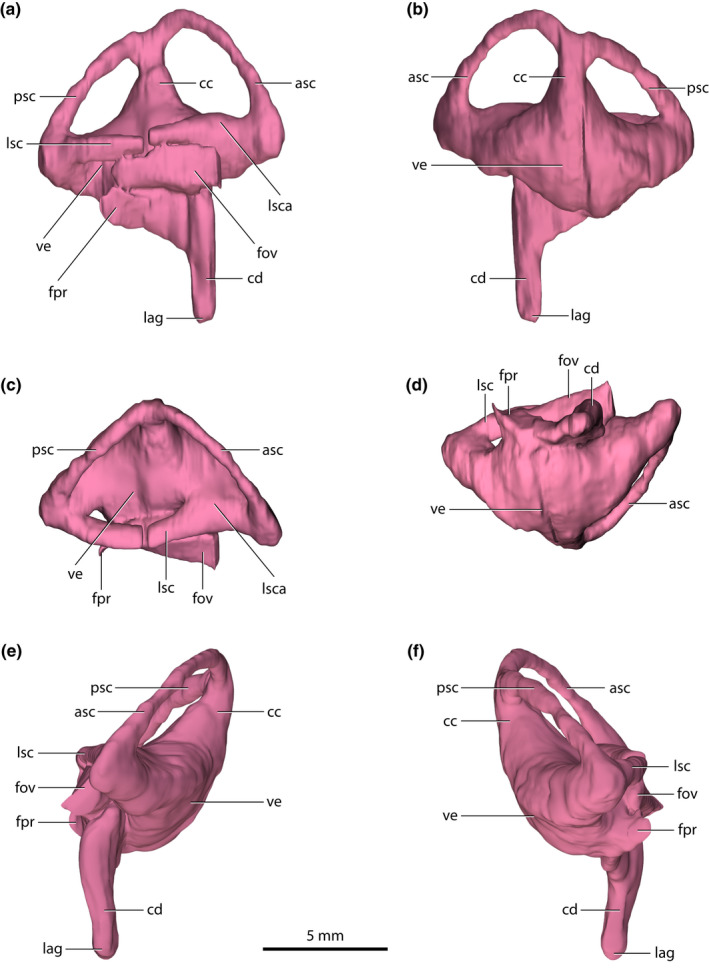
*Trilophosuchus rackhami* Willis, 1993, QMF16856, holotype. Right endosseous labyrinth in (a) lateral, (b) medial, (c) dorsal, (d) ventral, (e) anterior, and (f) posterior views. Abbreviations: asc, anterior semicircular canal (endocast); cc, common crus (endocast); cd, cochlear duct (endocast); fov, fenestra ovalis; fpr, fenestra pseudorotunda; lag, lagena (endocast); lsc, lateral semicircular canal (endocast); lsca, ampulla of lateral semicircular canal (endocast); psc, posterior semicircular canal (endocast); ve, vestibule (endocast).

**FIGURE 11:**
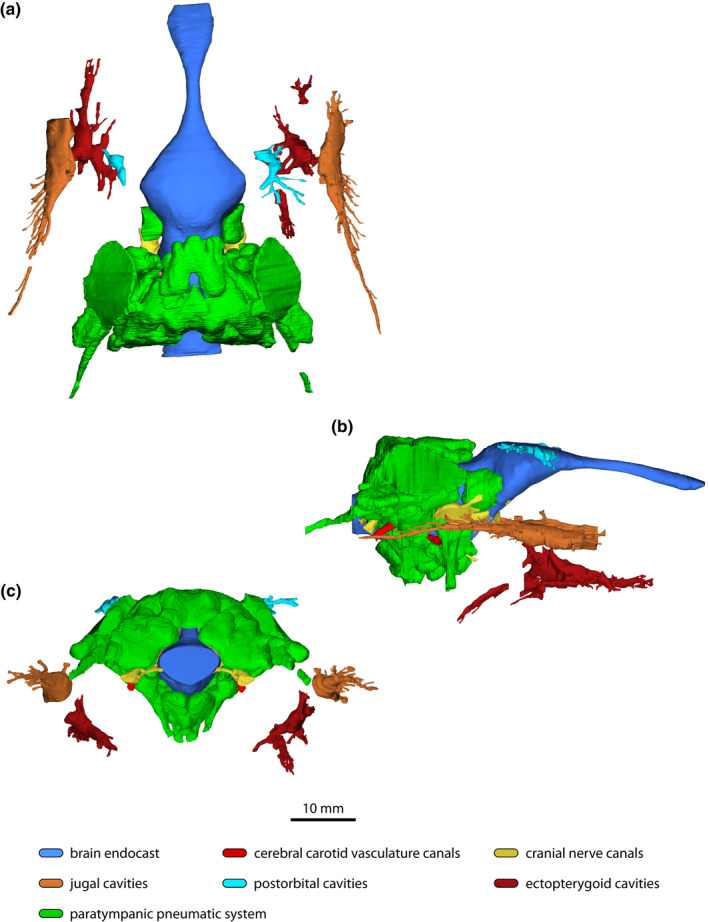
*Trilophosuchus rackhami* Willis, 1993, QMF16856, holotype. Non‐annotated digital models of the endocranial elements in (a) dorsal, (b) right lateral, and (c) posterior views. Note that the left jugal neurovascular cavity and both ectopterygoid vascular cavities could not be reconstructed in their entirety.

**FIGURE 12:**
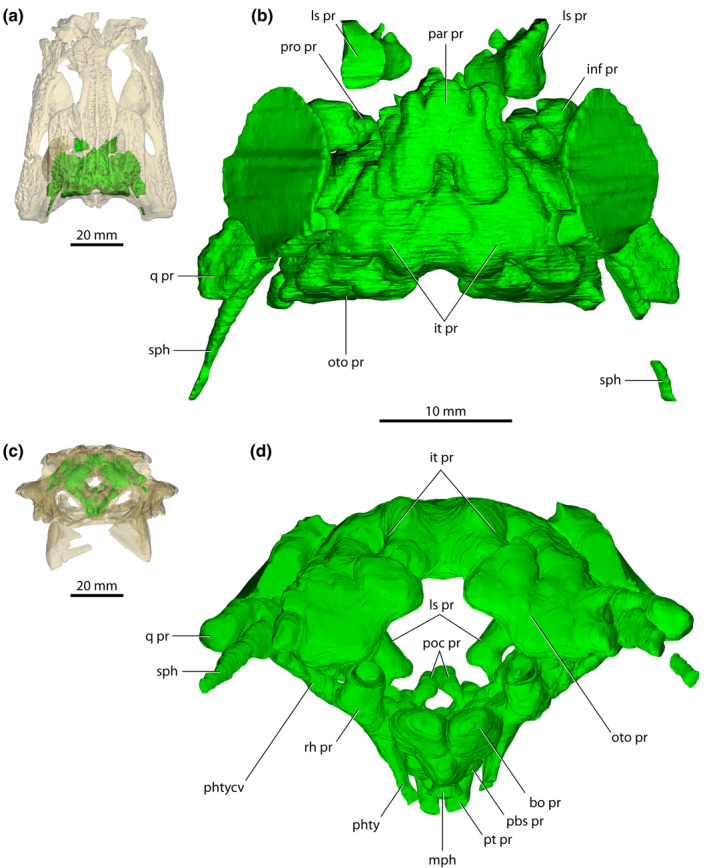
*Trilophosuchus rackhami* Willis, 1993, QMF16856, holotype. (a) Transparent digital model of the cranium in dorsal view, exposing the paratympanic pneumatic system. (b) Paratympanic pneumatic system in dorsal view. (c) Transparent digital model of the cranium in posterior view, exposing the paratympanic pneumatic system. (d) Paratympanic pneumatic system in posterior view. Abbreviations: bo pr, basioccipital recess; inf pr, infundibular recess; it pr, intertympanic recess; ls pr, laterosphenoid recess; mph, median pharyngeal canal; oto pr, otoccipital recess; par pr, parietal recess; pbs pr, parabasisphenoid recess; phty, pharyngotympanic canal; phtycv, pharyngotympanic (middle ear) cavity; poc pr, postcarotid pneumatic recess; pro pr, prootic facial recess; pt pr, pterygoid recess; q pr, quadrate recess; rh pr, rhomboidal recess; sph, siphonium.

**FIGURE 13:**
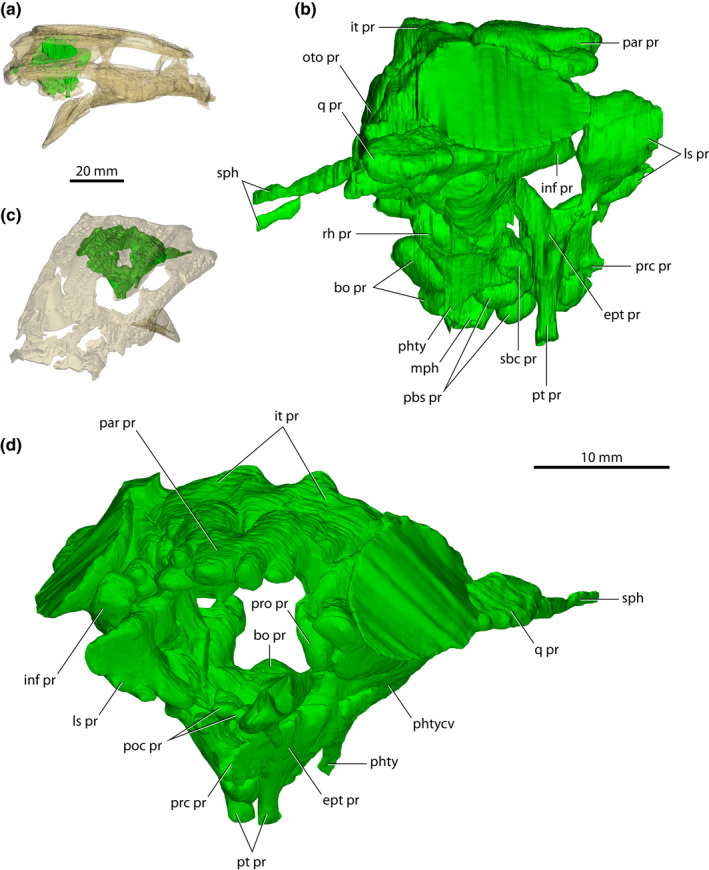
*Trilophosuchus rackhami* Willis, 1993, QMF16856, holotype. (a) Transparent digital model of the cranium in right lateral view, exposing the paratympanic pneumatic system. (b) Paratympanic pneumatic system in right lateral view. (c) Transparent digital model of the cranium in oblique anterodorsal view, exposing the paratympanic pneumatic system. (d) Paratympanic pneumatic system in oblique anterodorsal view. Abbreviations: bo pr, basioccipital recess; ept pr, recessus epitubaricus; inf pr, infundibular recess; it pr, intertympanic recess; ls pr, laterosphenoid recess; mph, median pharyngeal canal; oto pr, otoccipital recess; par pr, parietal recess; pbs pr, parabasisphenoid recess; phty, pharyngotympanic canal; phtycv, pharyngotympanic (middle ear) cavity; poc pr, postcarotid pneumatic recess; prc pr, precarotid pneumatic recess; pro pr, prootic facial recess; pt pr, pterygoid recess; q pr, quadrate recess; rh pr, rhomboidal recess; sbc pr, subcarotid recess; sph, siphonium.

The first descriptive study on *Trilophosuchus* was published by Willis et al. (1993). Since, *Trilophosuchus* has received little to no attention until this study and the study by Ristevski et al. (2022). Because QMF16856 is by far the most complete and best‐preserved specimen of the taxon, the aim of this study and that by Ristevski et al. (2022) is to provide detailed anatomical descriptions of the *T. rackhami* holotype based on information obtained via a high‐resolution μCT scan. The cranial osteology of QMF16856 as well as the phylogenetic relationships of *T. rackhami* are examined in detail by Ristevski et al. (2022). The focus of this study is exclusively on the neuroanatomy of *T. rackhami* (Figures 2, 3, 4, 5, 6, 7, 8, 9, 10, 11, 12, 13)—as such, there will be no discussion on its osteology and phylogeny.

Palaeoneurology or the study of the brain and nervous system of fossil taxa (particularly vertebrates) can offer crucial insights into the evolution, morphology, and even behavior of extinct organisms that cannot be deduced from osteomorphological investigations alone (Buchholtz & Seyfarth, 1999; Edinger, 1948; Hopson, 1979; Jerison, 1973; Walsh & Knoll, 2011). Aspects of crocodylomorph palaeoneurology were first investigated in the 19th century (Owen, 1842, 1850) and continued sporadically throughout the 20th century (e.g., Colbert, 1946a, 1946b; Edinger, 1938; Hopson, 1979; Yeh, 1958), though the frequency of palaeoneurological publications has increased drastically in the 21st century. With the advent of computed tomography and its increasing widespread availability to researchers, the past 14 years have witnessed a surge in papers that are dedicated either exclusively or partially on the palaeoneurology of crocodylomorphs (e.g., Blanco et al., 2015; Bona & Paulina Carabajal, 2013; Bona et al., 2013, 2017; Bowman et al., 2021; Brusatte, Muir, et al., 2016; Cowgill et al., 2021; Dumont Jr et al., 2020; Erb & Turner, 2021; Fernández et al., 2011; Fonseca et al., 2020; George & Holliday, 2013; Herrera et al., 2018; Herrera, 2015; Holliday & Gardner, 2012; Kley et al., 2010; Leardi et al., 2020; Melstrom et al., 2021; Pierce et al., 2017; Pochat‐Cottilloux et al., 2021; Puértolas‐Pascual et al., 2022; Ristevski et al., 2020a, 2021; Schwab et al., 2020; Schwab, Young, Herrera, et al., 2021, Schwab, Young, Walsh, et al., 2022; Schwab et al., 2022; Sereno & Larsson, 2009; Serrano‐Martínez et al., 2019a, 2019b, 2021; Sertich & O'Connor, 2014; Wilberg et al., 2021; Witmer & Ridgely, 2008; Witmer et al., 2008). Despite this, our understanding of crocodylomorph palaeoneurology is still in its relative infancy considering that the neuroanatomy for the vast majority of crocodylomorph taxa is yet to be investigated, including for the majority of Australian taxa.

Prior to this study, *Paludirex vincenti* Ristevski et al., 2020a was the only mekosuchine with available neuroanatomical data. Furthermore, this is the third study to explore palaeoneurological aspects of an extinct Australian crocodylian, after the aforementioned description for *P*. *vincenti* and that for the gavialoid *G. maunala* (see Ristevski et al., 2020a, 2020b, 2021). Because of the exceptional preservation of the *T. rackhami* holotype and the quality of the μCT data for the same, most of the specimens' neuroanatomical elements were digitally reconstructed. This neuroanatomical description is the most comprehensive yet for an extinct Australian crocodylomorph and provides an unprecedented insight into the palaeoneurology of Mekosuchinae.

## 2 MATERIALS AND METHODS

### 2.1 Micro‐computed tomographic scanning and 3D digital models

The *T. rackhami* holotype was scanned on March 20, 2019 at the Centre for Advanced Imaging at The University of Queensland using a Siemens Inveon multimodality PET‐CT imaging scanner. The scan parameters were set to 0.05116 mm of slice thickness, 80 kV voltage, 500‐μA current, and exposure time of 900 ms. The resulting dataset contains 2344 slices in DICOM format. The images acquired from the μCT scan were imported into the specialized 3D image processing software Mimics (Materialise NV, Belgium) at The University of Queensland and Flinders University. Digital models of the holotype cranium, as well as its isolated cranial bones and endocranial elements, were generated in several versions of Mimics (Mimics 21.0, 22.0, and 24.0), where the endocranial components were manually segmented using several tools from the Segment menu (primarily the lasso tool in Multiple Slice Edit). Afterwards the digital models of the cranial bones and endocranial elements were exported as STL files. The STL files were then imported into Materialise 3‐matic 16.0 in order to create the three interactive 3D PDF documents of the *T. rackhami* holotype specimen that are added as a supplement to this paper. These 3D PDFs were used in the creation of Figures 2, 3, 4, 5, 6, 7, 8, 9, 10, 11, 12, 13 of this paper, as well as the figures of *T. rackhami* in Supplemental Documents S1 and S2. The color codes of the digital endocranial models generally follow the examples set by Sereno et al. (2007) and Witmer et al. (2008), and in the case of the paratympanic pneumatic recesses, Dufeau (2011).

The interactive 3D PDF documents, alongside the individual STL files of the palaeoneurological elements as well as other supplementary data for this paper can be freely accessed at the Dryad Digital Repository via the following link: https://doi.org/10.5061/dryad.fbg79cnx4. Remaining supplementary material can be freely accessed at Zenodo via: https://doi.org/10.5281/zenodo.6968373. The raw μCT data for QMF16856 is available upon request from the Queensland Museum at MorphoSource: https://www.morphosource.org/concern/media/000431912.

### 2.2 Mensuration

The digitally reconstructed endocranial elements were measured in Mimics using the Distance and Angle tools from the Measure menu. The protocol for measuring the cephalic and pontine flexures of the brain endocast (Figure S2.1B) follows the instructions by Hopson (Hopson, 1979; see also Lautenschlager & Hübner, 2013 and Pierce et al., 2017). The measuring parameters in Table 1 (also Supplemental Document S4) are after Pierce et al. (2017), with several newly proposed parameters in this study (see Figures S2.1 and S2.5 in Supplemental Document S2 for measuring instructions).

**TABLE 1:** Brain endocast and endosseous labyrinth measurements for the holotype specimen (QMF16856) of *Trilophosuchus rackhami* Willis, 1993. The measurements are rounded to the nearest millimeter (mm). Exceptions are the estimates for the cephalic and pontine flexure angles, and the semicircular canal angles which are given in degrees, and those for the semicircular canal areas which are rounded to the nearest mm^2^. Original measuring parameters are after Pierce et al. (2017). Measuring parameters proposed herein for the first time are indicated with NEW. The estimate for the width of the olfactory bulb endocast is marked with an asterisk (*) due to the incompleteness of the bulbs in QMF16856. As such, the measurement for that parameter is that of their maximum reconstructed width. Instructions on how the measurements were obtained are provided in Figure S2.1 of supplemental document S2

Parameter	Estimate
Skull width between postorbitals, at the level of cerebral hemispheres	37
Cephalic flexure angle	136°
Pontine flexure angle	142°
Brain endocast, length	56
Olfactory apparatus (endocast), length	22
NEW Olfactory bulbs (endocast), width	7*
Brain endocast at cerebral hemispheres, width	17
NEW Brain endocast at cerebral hemispheres, height	10
NEW Midbrain region on brain endocast, width	10
NEW Hindbrain region on brain endocast, height	14
NEW Hindbrain region on brain endocast, width	9
Hypophyseal fossa, width	4
Hypophyseal fossa, height	2
Hypophyseal fossa, length	6
Endosseous labyrinth, height	13
Endosseous labyrinth, anteroposterior length	10
NEW Vestibular apparatus, height	8
NEW Common crus, height	3
Endosseous cochlear duct, height	6.5
Anterior semicircular canal area	8
Posterior semicircular canal area	4
Lateral semicircular canal area	2
NEW Angle between anterior and posterior semicircular canals	98°
NEW Angle between anterior and lateral semicircular canals	40°
NEW Angle between posterior and lateral semicircular canals	46°

### 2.3 Anatomical terminology

The neuroanatomical terminology in this study is mainly after Witmer et al. (2008). Anatomical terminology on the osteological components of the braincase follows Kuzmin et al. (2021). Likewise, the terminology and anatomical divisions of the cavities related to the paratympanic pneumatic system is after Kuzmin et al. (2021). As in Kuzmin et al. (2021), the digitally reconstructed cavities of the paratympanic pneumatic system are here referred as pneumatic recesses. The term diverticulum (plural, diverticula) is not used in referral to the skeletal pneumatic recess. Like Dufeau & Witmer (2015) and Kuzmin et al. (2021), I accept the definition of a pneumatic diverticulum as an air‐filled epithelial extension that occupies a recess. Therefore, a pneumatic diverticulum represents a soft‐tissue structure, and the bony cavity or depression created by the diverticulum is a pneumatic recess. This definition is also congruent with the study by Dufeau & Witmer (2015), where they used the term diverticula to describe epithelial outgrowths of their parent sinuses that create the bony recesses. Nearly all anatomical terms used here are in English instead of standard Latin. Traditional directional terms are used throughout, such as “anterior” and “posterior” as opposed to “rostral” and “caudal”.

Technically, the term endocast refers to a cast (be it digital or physical) of any internal, hollow cavity (Balanoff & Bever, 2020). In many palaeoneurological studies, the cavity that accommodated the brain (encephalon) is often called the cranial endocast and/or simply endocast (e.g., Bever et al., 2011; Handley & Worthy, 2021; von Baczko et al., 2018; Witmer & Ridgely, 2009), with some authors using the term brain endocast as well (e.g., Brown et al., 2020; Herrera et al., 2018; Leardi et al., 2020). Here, the term brain endocast is used in reference to the endocast of the cranial cavity that housed the brain. I use the term brain endocast out of convenience, and also to distinguish from the usage of the term endocast in reference to other reconstructions of non‐canal‐like structures (e.g., endocast of the trigeminal ganglion). However, it is crucial to acknowledge that the endocast of the cranial cavity that contained the brain does not provide an exact representation of that organ. As stated in many publications before (e.g., Bona et al., 2013; Hopson, 1979; Jerison, 1973; Jirak & Janacek, 2017; Kley et al., 2010; Leardi et al., 2020; Pochat‐Cottilloux et al., 2021; Ristevski et al., 2020a, 2021; Rogers, 1999; Serrano‐Martínez et al., 2019a, 2021; Sertich & O'Connor, 2014; Witmer et al., 2008), crocodylomorph brain endocasts do not reflect the precise morphology of the brain itself. This is because of the relatively thick dural envelope that surrounds the brain, such that the dura mater and its dural venous sinuses are significant contributors to the endocast's contours, proportions, and volume (Jirak & Janacek, 2017; Watanabe et al., 2019; Witmer et al., 2008). Additionally, the crocodylian brain undergoes ontogenetic changes that affect its shape (and consequently, the shape of the brain endocast; see Beyrand et al., 2019; Hopson, 1979; Hu et al., 2021; Jirak & Janacek, 2017; Lessner & Holliday, 2020) as well as ratio of brain volume versus endocast volume, where in juveniles the brain occupies more endocranial space than in mature individuals (Hopson, 1979; Hu et al., 2021; Hurlburt et al., 2013; Jirak & Janacek, 2017; Rogers, 1999; Watanabe et al., 2019; see Figure S2.3). Hence, the description is based on the brain endocast rather than the brain itself, which is obviously neither preserved nor accurately represented by the endocast of the fossil. While the brain endocast of *T. rackhami* outlines a rough, generalized shape of the brain's morphology, it is essentially a superficial representation of the dural envelope. Nevertheless, the brain endocast provides useful insight into the endocranial morphology (and to a lesser degree, the brain) of *T. rackhami* that contributes to the understanding of crocodylomorph neuroanatomy.

The brain has three major divisions – the forebrain (prosencephalon), midbrain (mesencephalon), and hindbrain (rhombencephalon). During embryonic development, the main subdivisions of the prosencephalon are the telencephalon and the diencephalon, whereas the subdivisions of the rhombencephalon are the metencephalon and the myelencephalon (Hopson, 1979; Lessner & Holliday, 2020; Romer, 1956; Wyneken, 2007). The main divisions can be roughly outlined on the brain endocast, which aids the description (Figure 6). Different bones of the cranium bound each division. Although QMF16856 is clearly not an embryonic specimen (see Ristevski et al., 2022, for a detailed ontogenetic assessment), the terms for the above‐mentioned subdivisions (particularly those of the hindbrain) are used to assist with topological orientation on the brain endocast merely out of convenience (i.e., “metencephalon region” is used instead of “anterior portion of hindbrain,” and “myelencephalon region” instead of “posterior portion of hindbrain”).

The term dural peak (sometimes called a median dural peak or dural expansion) has previously been used in descriptions for brain endocasts of dinosaurs and other archosauriforms (e.g., Brusatte, Averianov, et al., 2016; Paulina Carabajal, 2012; Sampson & Witmer, 2007; Stocker et al., 2016; Witmer & Ridgely, 2009). In this study, I apply the term dural peak to crocodylomorphs in referral to a prominent inflation of the dorsal longitudinal dural venous sinus over the hindbrain region of the brain endocast. As such, the usage of this term as applied herein only refers to a superficially similar looking structure on the brain endocast but does not imply homology between the dural peaks of crocodylomorphs and dinosaurs. Based on differences in the dural peak among crocodylomorphs, I differentiate between two morphotypes: an *acute dural peak* and a *blunt dural peak*. The proposed definitions for each morphotype are:

An acute dural peak is prominent inflation of the dorsal longitudinal dural venous sinus located on the dorsal surface of the brain endocast and concentrated over the hindbrain region. An acute dural peak is unpaired and culminates in an apex that appears sub‐triangular when the endocast is observed in lateral view. The sub‐triangular apex of the acute dural peak terminates on approximately the same level as, or dorsal to the dorsal‐most point of the forebrain region. A crocodylomorph that exemplifies this morphotype is *Trilophosuchus rackhami*. For visual reference, see Figures 5 and 6b, Figure S1.1C and S1.1D of this study.

A blunt dural peak is a prominent inflation of the dorsal longitudinal dural venous sinus located on the dorsal surface of the brain endocast and concentrated over the hindbrain region. A blunt dural peak is unpaired, with a lengthy, smoothly rounded, and blunt dorsal contour when the endocast is observed in lateral view. A crocodylomorph that exemplifies this morphotype is *Rukwasuchus yajabalijekundu* Sertich & O'Connor, 2014. For visual reference, see fig. 6c in Sertich and O'Connor (2014).

Further commentary on the dural peak morphotypes is given in the “Section 4” section below.

### 2.4 Comparative taxa

The neuroanatomical components of the *T. rackhami* holotype are compared with those of other crocodylomorphs, both extant and extinct. Endocranial data for most taxa are known from scans of single specimens, while a few of them (particularly extant taxa) come from multiple specimens of various ontogenetic stages. Not all taxa preserve all endocranial elements, and so for some only the brain endocast was used for comparison, while others provide information only on their endosseous labyrinths. Fewer have multiple elements available for comparative anatomy. The taxa considered for the comparative neuroanatomy and their sources are listed in Table S2.3 of Supplemental Document S2.

### 2.5 Institutional abbreviations mentioned in the text

FMNH, Field Museum of Natural History, Chicago, Illinois, USA.; QM, Queensland Museum, Brisbane, Queensland, Australia (F, fossil); ROM, Royal Ontario Museum, Toronto, Canada.

## 3 NEUROANATOMICAL DESCRIPTIONS OF THE *Trilophosuchus rackhami* HOLOTYPE SPECIMEN (QMF16856)

### 3.1 Brain endocast

An almost complete reconstruction of the brain endocast (Figures 2, 3, 4, 5, 6, 7, 8, 9 and 11, Figures S1.1 and S1.2) was achieved. Due to breakage on the parabasisphenoid, an anterior section of the hypophyseal fossa could not be rendered. Additionally, the incompleteness of the laterosphenoids (particularly, the anterior processes of the laterosphenoids; see Ristevski et al., 2022) prevents the complete reconstruction of the forebrain region of the endocast (more specifically, the portion corresponding to the endocast of the cerebral hemispheres). Otherwise, the rest of the reconstructed endocast faithfully maintains its dimensions and has no, or only negligible deformations.

The brain endocast occupies much of the specimen's endocranium. Measurements of the brain endocast are given in Table 1 (see also Supplemental Document S4 for comparative morphometrics). The brain endocast is bounded dorsally by the frontal (anterodorsally), parietal, and supraoccipital and otoccipitals (posterodorsally); laterally by the laterosphenoids (anterolaterally), prootics, and otoccipitals (posterolaterally); and, ventrally it is bounded by the parabasisphenoid (anteroventrally) and basioccipital (posteroventrally).

**FIGURE 14:**
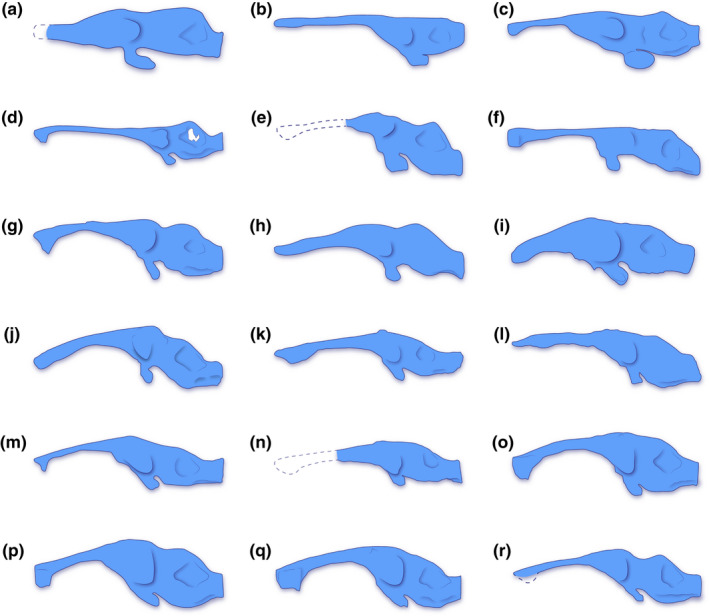
Brain endocast silhouettes of select crocodylomorphs, depicted in left lateral view. Dashed lines indicate hypothetical reconstructions of missing portions. Silhouettes not to scale. Brain endocast illustration of (a) *Almadasuchus figarii* Pol et al., 2013, (b) *Cricosaurus araucanensis* (Gasparini & Dellapé, 1976), (c) *Pelagosaurus typus* Bronn, 1841, (d) *Rhabdognathus aslerensis* Jouve, 2007, (e) *Araripesuchus wegeneri* Buffetaut, 1981, (f) *Campinasuchus dinizi* Carvalho et al., 2011, (g) *Rukwasuchus yajabalijekundu* Sertich & O'Connor, 2014, (h) *Sebecus icaeorhinus* Simpson, 1937, (i) *Simosuchus clarki* Buckley et al., 2000, (j) *Alligator mississippiensis* (Daudin, 1802), (k) *Crocodylus porosus* Schneider, 1801, (l) *Diplocynodon tormis* Buscalioni et al., 1992, (m) *Gavialis gangeticus* (Gmelin, 1789), (n) *Gunggamarandu maunala* Ristevski et al., 2021, (o) *Mecistops cataphractus* (Cuvier, 1824), (p) *Osteolaemus tetraspis* Cope, 1861, (q) *Tomistoma schlegelii* (Müller, 1838), and (r) *Trilophosuchus rackhami* Willis, 1993. For an expanded version of this figure see Figure S3.1 in supplemental document S3. The sources used to create these illustrations are given in Table S2.3 of supplemental document S2.

The brain endocast of *T. rackhami* has a form that generally resembles those of other mesoeucrocodylians, although its overall shape is not identical to any other crocodylomorph for which there is neuroanatomical data (see “Section 4” and Figure 14 and Figure S3.1). The endocast is relatively elongated and has moderate cephalic (the angle between the forebrain and midbrain) and pontine (the angle between the midbrain and hindbrain) flexures (Table 1). The forebrain division is well demarcated on the endocast, and presumably depicts some of its corresponding brain structures (such as olfactory apparatus and cerebra) more faithfully relative to the midbrain and hindbrain. This is presumed on the basis that in extant crocodylians the forebrain elements are more closely (but not firmly) appressed to the braincase than the midbrain and hindbrain elements (with the caveat in mind that in hatchlings and juveniles the brain is more closely appressed to the braincase than it is later in ontogeny; see Jirak & Janacek, 2017; Watanabe et al., 2019), and also because the dural envelope over the forebrain is comparatively thinner (Figure S2.3; Hopson, 1979; Watanabe et al., 2019). When observed in lateral aspect (Figures 5, 6 and 9, Figures S1.1C, S1.1D, S1.2C, and S1.2D), the brain endocast of *T. rackhami* has a notably sinusoidal dorsal contour. In lateral view, the dorsal contour of the endocast over the forebrain is relatively straight for almost its entire length before it starts descending into a prominent depression over the midbrain region. At the approximate midbrain‐hindbrain junction of the brain endocast, a “wave” begins to rise before it peaks acutely over the metencephalon region. Then, the “wave” drops steeply over the remaining portion of the metencephalon before it levels and becomes relatively straight for a short length (~6 mm) over the myelencephalon region. This markedly undulating dorsal contour of the brain endocast is induced by an impression of the dural venous sinuses that covered the brain. The dorsal contours of the forebrain region along with the midbrain (or, post‐cerebral) concavity, are largely an impression of the dorsal longitudinal dural venous sinus (for examples in other crocodylians see Porter et al., 2016 and Witmer et al., 2008). For most of its length, the dorsal longitudinal dural venous sinus is not a salient feature on the brain endocast, with its impression being faintly visible as a subtle and narrow dilation spread anteroposteriorly over the dorsal surface of the endocast.

The anterior portion of the forebrain region is represented by the olfactory apparatus, which includes the endocasts of the olfactory tract and olfactory bulbs. As reconstructed, this portion of the brain endocast is bounded exclusively by the frontal bone, with the descending processes of the frontal laterally bounding the olfactory tract for its entire length. The ventral surface of the olfactory apparatus is unossified. (In living crocodylians, there are two cartilaginous structures located ventral to the frontal – the trough‐like *planum supraseptale* and the interorbital septum, with the former sitting on top of the latter (Ali et al., 2008; de Iuliis & Pulera, 2011; Iordansky, 1973; Kuzmin et al., 2021). The *planum supraseptale* supports some of the forebrain elements ventrally, i.e., the olfactory apparatus and the cerebral hemispheres (Ali et al., 2008). This configuration composed of the aforementioned cartilaginous elements is expected to have been present in *T. rackhami* as well). The elongated olfactory apparatus comprises almost 40% of the total anteroposterior length of the endocast. When observed in lateral aspect, the olfactory apparatus is nearly straight and has a subtle anteroventral inclination. The olfactory tract endocast is widest at its posterior‐most portion (~10 mm mediolateral width), which is at the olfactory apparatus‐cerebral hemispheres junction. From there on, the olfactory tract gradually tapers anteriorly until it attains its narrowest point (~2 mm mediolateral width) a little over mid‐length of the olfactory apparatus. Then, the tract begins to gradually widen again until it reaches the olfactory bulb section. The olfactory bulbs are situated at the anterior‐most portion of the brain endocast. As reconstructed, the endocast of the olfactory bulbs is mediolaterally wide (~7 mm), but not particularly deep. Due to the relatively poor preservational condition of the prefrontal, including its descending process (i.e., the prefrontal pillar), the endocast of the olfactory bulbs could not be reconstructed to the point where they were bound by the prefrontal pillars. However, this is not to say that in a better‐preserved skull than that of QMF16856 the prefrontal pillars would not have bound the olfactory bulbs endocast. There is no median sulcus on the endocast between the olfactory bulbs.

The most voluminous portion of the forebrain is the endocast of the cerebral hemispheres. This is also the widest section of the entire brain endocast. This part of the endocast, once occupied by the cerebral hemispheres, is roughly spade‐shaped when observed from a dorsal aspect, and mildly tapering both anteriorly (i.e., toward the olfactory tract) and posteriorly (i.e., toward the midbrain portion). Anterodorsally, the endocast of the cerebral hemispheres is bounded by the ventral surface of the frontal, posterodorsally by the ventral (=endocranial) surface of the parietal, and laterally by the cerebral fossae of the laterosphenoids (preserved only on the right laterosphenoid). As mentioned above, this portion of the forebrain region is probably incompletely reconstructed due to breakage of the anterior processes of the laterosphenoids, which would have bounded the endocast anteriorly.

The hypophyseal (=pituitary) fossa along with its associated cavernous dural venous sinus could not be fully reconstructed, as it is missing its anterior half which in life would have hosted the anterior part of the pituitary as well as the infundibulum, optic chiasma, and the roots of the optic nerves. What could be reconstructed of the hypophyseal fossa is bound entirely by the parabasisphenoid. A hypothetical reconstruction of the anterior missing section of the fossa is provided, however (Figures 3a, 4b and 5). The hypophyseal fossa is inclined and has a posteroventral orientation. Continuing posteriorly from the hypophyseal fossa are the cerebral carotid vasculature canals.

The midbrain region of the brain endocast is poorly defined and is not marked by any stand‐out features. This part of the endocast is dorsally bound by the endocranial surface of the parietal, ventrally by the dorsal (=endocranial) surface of the parabasisphenoid, and laterally by the laterosphenoids. The optic lobes (optic tectum) of the brain were present in this region, yet their impressions are not clearly discernable on the endocast. The most peculiar feature of the midbrain region is the deeply concave surface dorsally on the endocast. In addition, the midbrain region has a concave ventral contour when observed in lateral aspect, posterodorsal to the hypophyseal fossa. Thus, the midbrain region of the endocast appears dorsoventrally constricted when viewed from a lateral aspect.

The anterior portion of the hindbrain (corresponding to the metencephalon region) is dorsally bound by the supraoccipital, laterally by the prootics and smaller anterior sections of the otoccipitals, and ventrally by the parabasisphenoid. In life, the cerebellum and pons were the major brain structures located in the metencephalon region of the endocast. Much of the lateral surfaces of the metencephalon region are constricted by the otic bullae (formed by the prootics, supraoccipital, and otoccipitals). Note that this lateral constriction is reflected only on the brain endocast and would not imply equivalent constriction on the brain itself. The metencephalon region is mainly characterized by a prominent dural peak, which is responsible for the above‐described “wave” on the dorsal contour of the endocast. When observed from a lateral aspect, the acute dural peak over the metencephalon region has a somewhat pointed apex, thus appearing as a sub‐triangular inflation. Although the acute dural peak roughly corresponds to a location dorsal to where the cerebellum was situated, the dural peak is not an impression of the cerebellum itself. Instead, this acute peak is caused by impressions of the dorsal and occipital longitudinal dural venous sinuses (the latter being a posterior extension of the dorsal longitudinal dural venous sinus; Porter et al., 2016; Witmer et al., 2008). The apex of the acute dural peak is nearly on the same plane as the dorsal‐most point of the forebrain region, being only slightly lower (<1 mm). The impression of the occipital dural venous sinus is the most conspicuous of the dural venous sinuses on the dorsal surface of the endocast. In dorsal view, the acute dural peak is mediolaterally widened at the (approximate) anterior section of the occipital dural venous sinus. Faint anterolateral widenings near the midbrain‐hindbrain junction give away the impressions of the transverse sinuses. Arising from the apex of the acute dural peak are the paired canals for the external occipital veins (Figures 7, 8 and 9, Figures S1.2A and S1.2C–S1.2F). These vein canals are slender (~0.2 mm in diameter) and short (~2.6 mm for the left canal; ~3.5 for the right canal). Both canals are oriented posterodorsally, and pass through the supraoccipital with each canal piercing the anterior lamina of the bone via a tiny foramen that has a length of ~0.2 mm along its minor and ~0.4 mm along its major axis. There are no impressions of the floccular lobes (cerebellar auricles) on the brain endocast.

The posterior portion of the hindbrain on the endocast (corresponding to the myelencephalon region) contained the remainders of the medulla oblongata, ventral longitudinal dural venous sinus, and the occipital sinus. It is bounded by the otoccipitals dorsally and laterally, and the basioccipital ventrally. The sub‐oval cross‐sectional outline of the myelencephalon region mirrors that of the foramen magnum. Visible on the ventral surface of the myelencephalon region is a swelling that represents an impression of the ventral longitudinal dural venous sinus. The ventral longitudinal dural venous sinus impression is easily distinguishable ventrally on the brain endocast and is spread along the ventral surface of the entire hindbrain region. The impression of the ventral longitudinal dural venous sinus is widest on the ventral surface of the metencephalon region (~6.5 mm mediolateral width), while it is narrowest (and comparatively fainter) along the ventral surface of the myelencephalon region (~3 mm mediolateral width).

### 3.2 Cranial nerve and vasculature endocasts/canals

Most cranial nerve endocasts/canals of QMF16856 were reconstructed. The endocasts of the cranial nerves and cranial vasculature are tracings of the canals that the nerves, veins, and arteries carved through the skull when the animal was alive. Reliable reconstruction for the optic nerves endocast (cranial nerves II) was not possible, however. In crocodylians, the osteological boundaries/rims for the optic nerve endocast/canal are located anteromedially on the braincase, or more specifically, they are bounded by the anterior processes of the laterosphenoids (Iordansky, 1973; Kuzmin et al., 2021; Lessner & Holliday, 2020; Witmer et al., 2008). As described by Ristevski et al. (2022), the left laterosphenoid of QMF16856 is largely broken, whereas the morphology of the right laterosphenoid indicates that the anterior of the braincase was probably largely unossified. Considering these factors, it was not possible to reconstruct the optic nerves endocast. Similarly, reliable reconstructions of the oculomotor nerves endocasts (cranial nerves III) was difficult due to the poorly defined margins of the oculomotor foramina (even though the margins of the oculomotor foramina are partially discernable). The related foramina for the reconstructed cranial nerve and vasculature endocasts/canals are described by Ristevski et al. (2022).

#### 3.2.1 Trochlear (CN IV) nerve canal (tentative)

A short (~1 mm) canal that excavates the right laterosphenoid anteroventrally is tentatively identified as pertaining to the trochlear nerve (cranial nerve IV; Figures 7a, 8b and 9a). Due to its small dimensions, little can be described of this canal. It originates from the ventral surface of the endocast for the right cerebral hemisphere and has a diameter of ~0.5 mm. The left counterpart of this canal is not preserved due to breakage on the left laterosphenoid.

#### 3.2.2 Trigeminal (CN V) nerve endocasts and canals

The largest cranial nerve endocasts and canals are those for the trigeminal nerves (cranial nerves V; Figures 7, 8, 9 and Figure S1.2). Each trigeminal canal offshoots laterally from the brain endocast. The root of both the left and right canal is stout (~3 mm) and extends laterally for a short length (<1 mm) before it exits the braincase through the trigeminal foramen and expands into the endocast of the trigeminal (Gasserian) ganglion. The endocast of the trigeminal ganglion sits outside the braincase within the trigeminal fossa (or, Meckel's cave; see George & Holliday, 2013) that is bound by the laterosphenoid anteriorly, prootic posteriorly, and quadrate dorsolaterally. Extending from the endocast of the trigeminal ganglion are four canals, two of which are for major branches of the nerve—the canal for the ophthalmic branch (CN V_1_), and the canal for the maxillary branch (CN V_2_). Additionally, smaller branches—the canal for the supraorbital nerve, and the canal for the tympanic branch of the trigeminal nerve, excavated the other two canals. The trigeminal nerve has a third major branch, which is the mandibular (CN V_3_), although a distinct canal for the mandibular branch is not traceable. Nevertheless, the approximate location for the root of the mandibular branch can be inferred based on its corresponding location in extant crocodylians (George & Holliday, 2013; Holliday & Witmer, 2009; Lessner & Holliday, 2020; Witmer et al., 2008), which would be near the posterolateral section of the trigeminal endocast (Figures 8b and 9).

On both the left and right trigeminal endocast, the largest reconstructed canal is that for the ophthalmic branch of the maxillary nerve. This canal commences from the anteroventral section of the trigeminal ganglion endocast and extends anteroventrally for approximately ~4 mm through the cavum epiptericum of the laterosphenoid (and is bounded laterally by the lateral bridge of the laterosphenoid). The reconstructed cross‐sectional outline of each ophthalmic branch canal is elliptical (~2.7 mm along major axis length, and ~1.2 mm along minor axis length).

The canal for the maxillary branch was reconstructed for only a short length on both sides (anteroposterior length of ~2 mm). Each canal was reconstructed based on the impression of the groove for the maxillary nerve branch that is discernable on the lateral surface of the laterosphenoid, dorsal to the lateral bridge.

The canal for the supraorbital nerve (which is the first branch for the maxillary nerve) is somewhat asymmetrical between the left and right side. This canal is better defined on the left trigeminal endocast, as it commences immediately dorsal to the canal for the maxillary branch and extends in an anterodorsal direction. A faint medial curvature also characterizes the supraorbital nerve canal. On the left side, the canal courses through the laterosphenoid and exits the bone through the small supraorbital nerve foramen. The left canal for the supraorbital nerve is ~2.6 mm long and has a diameter of ~0.5 mm. The supraorbital nerve canal on the right side is not as well defined, particularly laterally as it is not enclosed by the laterosphenoid, and there is no supraorbital nerve foramen either. There is no apparent breakage on the right laterosphenoid around the area where the supraorbital nerve canal passes. As reconstructed, the supraorbital nerve canal on the right side is ~2.8 mm long and has a diameter between ~0.4 and 1 mm. Other than this discrepancy, the right canal is also located immediately dorsal to the maxillary branch canal and has an anterodorsal orientation with a mild medial curvature.

The canals for the tympanic branches of the trigeminal nerves and sympathetic nerves are the slenderest components of the trigeminal nerve endocasts. Each tympanic branch commences from the dorsal surface of the trigeminal ganglion endocast and has a largely dorsal trajectory with a modest posterior orientation. Each canal for the tympanic branch pierces the prootic through a small foramen (~0.3 mm in diameter) and passes through a narrow passage between the quadrate‐prootic sutures. Both left and right tympanic branch canal were reconstructed for a length of ~3 mm and each has a diameter of ~0.4 mm.

#### 3.2.3 Abducens (CN VI) nerve canals

The canals for the abducens nerves (cranial nerves VI; Figures 7a, 8b and 9a) originate from the ventral surface of the brain endocast and excavate the anterodorsal half of the parabasisphenoid. Due to damage at the left anterolateral side of the parabasisphenoid, the right canal was reconstructed for more of its length than the left. Both abducens canals are quite narrow (~0.6 mm in diameter), with the right canal having a reconstructed anteroposterior length of ~4.6 mm while the left is reconstructed for an anteroposterior length of ~2.6 mm. These canals are straight for their entire reconstructed lengths and lack obvious curvature. They flank the hypophyseal fossa dorsolaterally.

#### 3.2.4 Facial (CN VII) nerve canals

Partial reconstruction for the canals of the facial nerves (cranial nerves VII; Figures 7, 8, 9) was possible on both left and right side. Thus, the canals for the two branches of the facial nerves—the hyomandibular and palatine—were partially reconstructed.

Each canal for the hyomandibular branch of the facial nerve is situated along the lateral surface of the brain endocast and pierces the prootic via a conspicuous foramen. Both hyomandibular canals project dorsolaterally from the brain endocast at an angle of ~27°. The hyomandibular branch canals are short (~1.85 mm for the right and ~1.5 mm for the left canal) and have a diameter of ~0.7 mm. On the endocast, each canal for the hyomandibular branch is exposed immediately ventral to the impression of the ampulla for the lateral semicircular canal.

The canals for the palatine branches of the facial nerves could not be reconstructed for their entire lengths, and it was not possible to trace them to their roots. Nevertheless, the reconstructed portions for these branches are long and slender. As reconstructed, the palatine branch canals are carving their paths through the parabasisphenoid, and both left and right canal have a diameter of ~0.5 mm. The right canal was reconstructed for a length of ~8 mm, and the left for a length of ~6 mm. Beginning from their distal (preserved) ends, the palatine branch canals extend anteroventrally and gradually begin to converge until they attain a relatively constant distance from each other approximately mid‐length. Once they attain a relatively constant distance, they continue their anteroventral descent through the parabasisphenoid. Compared to the right, the left canal has a slightly more accentuated ventral orientation.

#### 3.2.5 Vestibulocochlear (CN VIII) nerve endocast

The endocast(s) for the vestibulocochlear nerve (cranial nerve VIII; Figures 7b, 8a and 9a) was reconstructed only on the right side, as the damage on the left prootic has obliterated the foramina and canals for these nerves on the left. Although not reconstructed due to their shallowness, it should be noted that each prootic also bounds a foramen for a third branch of the vestibulocochlear nerve, which is that for the posterior branch of the vestibulocochlear nerve (see Ristevski et al., 2022). As reconstructed, the endocast for the vestibulocochlear nerve on the right side is paired and passes through two foramina that pierce the medial surface of the prootic at the ventral margin of the otic bulla. Additionally, it originates on the lateral surface of the hindbrain region of the brain endocast, dorsolateral to the canal for the hyomandibular branch of the facial nerve. One of the reconstructed branches for the vestibulocochlear nerve corresponds to the anterior branch and the other for the macula sacculi branch of the nerve. Both reconstructed branches are truncated and have small diameters, with the anterior branch being marginally larger than the macula sacculi branch (~0.6 mm in anteroposterior length and ~0.4 mm in transverse width for the macula sacculi branch endocast; ~0.7 mm in anteroposterior length and ~0.6 mm in transverse width for the anterior branch endocast). Further reconstruction was not possible as after their brief lengths, the canals for the branches of the vestibulocochlear nerve cease to be traceable within the endocranial space. The macula sacculi branch is reconstructed to the point where it “abuts” the vestibule of the endosseous labyrinth anteromedially, whereas the anterior branch is reconstructed to the point where it ventromedially “abuts” the endocast of the ampulla of the lateral semicircular canal.

#### 3.2.6 Common canals for cranial nerves IX–XI and their accompanying vessels

After the trigeminal, the second largest cranial nerve canals are the common canals for cranial nerves IX (glossopharyngeal), X (vagus), XI (accessory), and their associated vessels (Figures 7, 8, 9). The accessory nerve (cranial nerve XI) does not originate in the skull and does not target any cranial structures (it targets cervical musculature instead; Lessner & Holliday, 2020). Nevertheless, in living crocodylians, the accessory nerve passes through the head along with the glossopharyngeal and vagus nerves (Kuzmin et al., 2021). Hence, the common canal for CN IX–XI of *T. rackhami* is also interpreted as the accessory nerve having passed through it.

Virtually complete reconstruction was possible for both the left and right CN IX–XI canal. Each canal originates from the lateral surface of the hindbrain and offshoots posterolaterally and ventrally. Medial‐most at its point of origin, each CN IX–XI canal conforms to the dimensions of the metotic foramen on the otoccipital and gradually tapers laterally until it attains a tubular form that continues to course posteroventrally through the otoccipital. Both canals have a length of ~8 mm and a diameter (measured approximately halfway along the length of each canal) of ~1 mm. As reconstructed, the diameter of each CN IX–XI canal is constant for most of its length until it reaches its exit externally on the otoccipital, through the common external foramen for CN IX–XI. There, the CN IX–XI canals widen significantly according to the mediolateral width of the foramen.

Anterolaterally, each CN IX–XI canal gives rise to a prominent outgrowth corresponding to the canal for the tympanic branch of the glossopharyngeal and vagus nerves. Each tympanic branch is curved dorsolaterally and has a length of ~1.6 mm and a thickness of ~0.5 mm.

#### 3.2.7 Hypoglossal (CN XII) nerve canals

Three canals on each side, an anterior, a middle, and a posterior canal (Figures 7b, 8 and 9) represent the hypoglossal nerves (cranial nerves XII). The hypoglossal canals originate from the posterior portion of the hindbrain region from where they offshoot laterally and have mild posteroventral curvature. The otoccipitals host these canals for their entire lengths and exit the braincase posteriorly via their respective hypoglossal foramina on the external surfaces of the otoccipitals. The anterior hypoglossal canals are the slenderest of the three, with each having a thickness of ~0.2 mm. Both left and right anterior canal have a length of ~4 mm. The middle hypoglossal canals are positioned more closely to the anterior than the posterior canals. Each middle hypoglossal canal has a thickness of ~0.4 mm and length of ~4 mm. The anterior and middle hypoglossal canals mirror each other in that they have more accentuated ventral curvatures than the posterior canals. The posterior hypoglossal canals are the posterior‐most cranial nerve canals. Compared to the anterior and middle, the posterior hypoglossal canals are slightly shorter (~3 mm) and thicker (0.7–1 mm).

#### 3.2.8 Cerebral carotid vasculature canals

The canals for the cerebral carotid vasculature (Figures 7, 8, 9, 11 and Figure S1.2) are extensive, spreading for a length of ~16 mm, and each canal has a diameter of ~1.5 mm. In life, these canals would have hosted the cerebral carotid arteries and veins. Due to damage within the braincase, certain intermediate sections of the canals could not be reliably reconstructed, although more of the left canal could be reconstructed as opposed to the right. Anteriorly, each cerebral carotid vascular canal can begin to be traced from its respective anterior carotid foramen within the parabasisphenoid. Both canals are roughly parallel to each other and straight for a brief length of ~1.6 mm before they start to diverge and take a sharp lateral course. Then, both canals begin to curve strongly in a dorsolateral direction as they exit the parabasisphenoid through conspicuous foramina found posterolaterally on the bone. As the middle sections of both canals could not be traced (with a partial exception on the left), the exact trajectory of the path the canal took is uncertain for a relatively short length. The remainder of each canal was fully reconstructed from the point where they enter the otoccipital through a conspicuous foramen located anteroventrally on the element. Within the otoccipitals, the canals have mildly undulating paths, so that they strongly curve posteroventrally and only faintly laterally. Each canal exits the braincase through its respective posterior carotid foramen on the external surface of the otoccipital.

### 3.3 Additional vascular cavities and canals

The μCT data reveals that the postorbitals, jugals, and ectopterygoids also have substantial hollow cavities (Figures 2 and 11 and Figures S1.9–S1.11). The cavities and/or canals within these elements are non‐pneumatic and do not communicate with any segment of the paratympanic pneumatic system. Likewise, these cavities did not establish communication with the paranasal sinuses (which could not be reconstructed in the specimen due to the largely missing snout). Rather, the nature of these cavities is vascular and/or neurovascular.

#### 3.3.1 Vascular cavities of the postorbitals

The postorbitals of *T. rackhami* house canals that, in life, were likely traversed by vascular vessels such as the postorbital veins (as in extant crocodylians; see Porter et al., 2016; Sedlmayr, 2002; Figures 2b,c,f,g and 11 and Figure S1.9). Each postorbital cavity is primarily concentrated around the anterior portion of the element, thus excluding the posterior process of the postorbital from receiving a vascular branch. An asymmetry is evident between the branching of the right and left postorbital cavity, as the cavity within the right postorbital branches out into multiple canals. The canals exit via several foramina that pierce the postorbitals. These foramina are found on the orbital lamina of the postorbital, and posteromedially just posterodorsal to the socket that received the capitate process of the laterosphenoid. Additional foramina that serve as exits for the postorbital vascular canals are visible more posteriorly along the lateral margin of the postorbital.

#### 3.3.2 Neurovascular canals of the jugals

Internally, each jugal is hollowed out by an elongated neurovascular canal (Figures 2b,c,f,g and 11 and Figure S1.10). In crocodylians, the neurovascular canal of the jugal is related to the jugal subdivision/subbranch of the maxillary branch of the trigeminal nerve that innervates the skin of the face (Lessner & Holliday, 2020). The better preservation of the right jugal allowed for a virtually complete reconstruction of its neurovascular canal. Although much of the canal inside the left jugal could be reconstructed, it is nonetheless incomplete, particularly its anterior section. Nevertheless, the reconstructed canals of both jugals have a relatively consistent morphology. Due to its complete state, this description will be based on the neurovascular canal of the right jugal. The neurovascular canal of the right jugal extends internally for almost the entire length of the element (~39 mm). Its most voluminous portion is a central cavity located near the anterior process of the jugal, ventral and slightly anterior to the ascending process. The ascending process of the jugal is not affected by these canals, except for a single relatively small foramen found laterally at its base that serves as an exit for one of the branches that arises from the central cavity. Posterior to the level of the ascending process, the central cavity begins to strongly taper within the posterior process of the jugal. The posterior extent of the canal closely approaches but does not reach the terminal point of the jugal, and is therefore discontinuous from the quadratojugal. Numerous smaller canals branch out in a lateral and laterodorsal direction from the central cavity/canal toward the neurovascular foramina that adorn the external lateral surface of the jugal. These branching canals are most abundant within the posterior process of the jugal, while comparatively fewer canals branch out of the central cavity within the anterior process.

#### 3.3.3 Vascular cavities and canals of the ectopterygoids

Contained within the ectopterygoids of the *T. rackhami* holotype are cavities that also branch out into multiple canals (Figures 2b,c,f,g and 11 and Figure S1.11). Neither the right nor left ectopterygoid cavity and associated canals were reconstructed in their entirety due to incompleteness and/or damage that has affected each element. Still, substantial portions of these cavities are available for assessment. The most voluminous part of each ectopterygoid cavity is located laterally near the ascending process of the bone. As better represented by the left ectopterygoid, the cavity branches out into several canals anteriorly, posteriorly, but also laterally and dorsally. Some of these canals ramify into additional smaller canals. A posterolateral branch extends within the body of the ectopterygoid, although it does not occupy the entirety of that section of the element. Posteriorly, the right ectopterygoid develops a canal (that is further subdivided into several small canals) that extends within the descending process, although it does not reach its posterior tip. The ectopterygoid canals approach the lateral margins of the bone but do not reach the lingual alveolar walls that are formed by the ectopterygoids. Several small foramina dispersed over the dorsal and ventral surfaces of the ectopterygoids serve as exits for the canals that are spread internally in the bones. The canals within the ectopterygoids are probably vascular in nature because: they are isolated from other cranial cavities, be it pneumatic or vascular (even though they are located near the jugal neurovascular canals, they do not communicate with them); and, their branching patterns are more consistent with the distribution of vascular canals rather than pneumatic recesses created from diverticular expansion.

### 3.4 Endosseous labyrinths

Endocasts of both the left and right endosseous labyrinth of the inner ear (sensu Witmer et al., 2008) were reconstructed, with the left labyrinth being less complete (Figures 2d,h and 10 and Figures S1.2–S1.4). Due to the substantial damage on the capsular portion of the left prootic, part of the left vestibular apparatus (an anteromedial section of the vestibule, and a segment of the anterior semicircular canal) could not be reconstructed in its entirety. Even though the right endosseous labyrinth is virtually complete, it still suffers a minor deformation. There is a subtle dorsal displacement (roughly 0.2–0.3 mm) of the right prootic relative to its posterior contact with the right otoccipital. This displacement affects the way the right endosseous labyrinth is reconstructed, and thus its lateral semicircular canal is slightly deformed. Other than these small discrepancies, the reconstructions of the endosseous labyrinths are faithful to the morphology of these endocranial elements. As both endosseous labyrinths are symmetrical and without any notable morphological differences, the description is based primarily on the more complete right endosseous labyrinth.

In living animals, the endosseous labyrinth contains the membranous labyrinth that is filled with endolymphatic fluid, with the space between the membranous and bony labyrinth being filled by perilymph (Baird, 1960, 1970; de Burlet, 1929; Hullar, 2006; Pfaff et al., 2019). Each endosseous labyrinth is bound by the prootic anteriorly, otoccipital posteriorly, supraoccipital dorsally, and (with a small contribution) parabasisphenoid ventrally. Relative to the brain endocast, the endosseous labyrinths are located at the hindbrain region. The dorsal component of the endosseous labyrinth is the vestibular apparatus, which is composed of the three semicircular canals and the vestibule, whereas the ventral component of the labyrinth is the endosseous cochlear duct. The vestibular apparatus is bounded by the otic bulla (formed by the prootic anteriorly, supraoccipital dorsally, and otoccipital posteriorly) and has a sub‐pyramidal shape. The vestibule (which in life contained the utriculus and sacculus) is the most voluminous component of the endosseous labyrinth and forms the base of the vestibular apparatus. The dorsoventral height of the vestibular apparatus is ~8 mm, or 61.5% of the total endosseous labyrinth height. Rising dorsally from the (approximate) medial portion of the vestibule is the common crus of the anterior and posterior semicircular canals (sometimes called the crus communis or crus commune). Each common crus is widest at the “base” (~3 mm in cross‐sectional diameter) and gradually tapers dorsally (~1 mm in cross‐sectional diameter at its narrowest dorsal portion). The common crus of *T. rackhami* is relatively tall, with its dorsoventral height being ~3 mm. Virtually all of the common crus is bound by the supraoccipital, passing within the utricular recess of the bone.

The semicircular canals are well developed, and all three of them have subequal diameters (0.5–0.8 mm). The anterior and posterior semicircular canals meet at the dorsal tip of the common crus. Since the anterior semicircular canal is the most prominent, the vacant area between it and the common crus is twice that between the common crus and the posterior semicircular canal (Table 1). Both the anterior and posterior semicircular canal have a gentle medial curvature toward the dorsal tip of the common crus. The dorsal tip of the anterior semicircular canal is on a more dorsal level relative to the posterior, which accentuates the sub‐pyramidal appearance of the vestibular apparatus when observed in lateral and medial views. The dorsal section of the anterior semicircular canal courses through the supraoccipital, while the anteroventral through the prootic. Like the anterior, the dorsal section of the posterior semicircular canal also passes through the supraoccipital, but its posteroventral section is bound by the otoccipital. When observed from a dorsal aspect, the anterior and posterior semicircular canal form an angle of approximately 97.5°. Additionally, the angles formed by the anterior and lateral, and posterior and lateral semicircular canals as seen in dorsal view range from 40° to 46° (Table 1). The lateral semicircular canal courses through the otoccipital (posterior half) and prootic (anterior half). Based on the complete left lateral semicircular canal, it is evident that it displays a mild lateral curvature when viewed in dorsal and/or ventral view, and there is also a subtle anterodorsal bend toward the ampulla of the lateral semicircular canal. The impression of the ampulla of the lateral semicircular canal can be inferred on both labyrinths as a dilation that is located immediately anterior to the lateral semicircular canal. The impressions of the ampullae for the anterior and posterior semicircular canals are not clearly represented on the endosseous labyrinths. The vacant space between the lateral semicircular canal and the vestibule is smaller in comparison to the space between the vestibule and the anterior and posterior canals.

The fenestra ovalis (=fenestra vestibuli of Witmer et al., 2008) was reconstructed almost entirely on the right endosseous labyrinth. In contrast, the fenestra ovalis on the left appears smaller since its posterior boundary by the otoccipital is not as complete, and thus the dimensions of the fenestra vestibuli on the right endosseous labyrinth are more accurate. The fenestra ovalis is found on the lateral side of each endosseous labyrinth, immediately ventral to the lateral semicircular canal. The fenestra pseudorotunda (=fenestra cochleae of Witmer et al., 2008) is partially reconstructed on both the left and right sides. What is reconstructed of the fenestra pseudorotunda is located immediately posteroventral to the fenestra ovalis.

The endosseous cochlear duct extends lateroventrally from the vestibular apparatus and gradually tapers toward its ventral apex, where the lagena would have been. The endosseous cochlear duct is relatively straight for its entire length, lacking pronounced bending. The ventral‐most portion of the endosseous cochlear duct (the lagenar portion) sits in a depression – the cochlear recess – on the parabasisphenoid. In *T. rackhami*, the floor of the cochlear recess is bound solely by the parabasisphenoid, without contribution from the basioccipital.

### 3.5 Paratympanic pneumatic system

The holotype of *T. rackhami* has a considerably pneumatized cranium, such that most preserved bones are occupied by extensive pneumatic cavities (Figures 2b,c,f,g and 11, 12, 13). Thus, the bones of the chondrocranium (laterosphenoids, prootics, parabasisphenoid, basioccipital, otoccipitals, and supraoccipital), the splanchnocranium (quadrates, which are the only bones of the splanchnocranium preserved in QMF16856), and the parietal (part of the dermatocranium) are invaded by the paratympanic pneumatic recesses.

In crocodylians, the complex paratympanic pneumatic system is consisted of two primary pneumatic expansions, which are the pharyngotympanic sinus (tympanic or middle ear cavity) and the median pharyngeal sinus (Dufeau & Witmer, 2015; Kuzmin et al., 2021). Relative to the brain endocast, the paratympanic sinuses are mainly concentrated around the midbrain and hindbrain regions (Figures 2b,c,f,g, and 11). Early in crocodylian ontogeny, some elements of the paratympanic pneumatic system have no communication between them, although later (and before attaining skeletal maturity), the sinuses establish multiple connections (Dufeau & Witmer, 2015; Kuzmin et al., 2021). In the *T. rackhami* holotype, these communications are already established and the pharyngotympanic and median pharyngeal sinuses are confluent, which makes precise delineation of the boundaries between them difficult. Many diverticular expansions of the sinuses have intricate shapes, and some give rise to smaller accessory recesses.

The parabasisphenoid is internally occupied by a complex network of pneumatic recesses, most notable of which is the median pharyngeal recess. The median pharyngeal recess may be subdivided into the median pharyngeal canal (or median pharyngeal tube) and the parabasisphenoid pneumatic recesses (Figures 12d and 13b). The median pharyngeal pneumatic canal is short and stout (~3 mm dorsoventral length; ~3 mm diameter). It passes between the basioccipital and parabasisphenoid and has a slight posteroventral (as opposed to fully vertical) orientation. The median pharyngeal pneumatic canal exits the basicranium through the median pharyngeal tube foramen (sensu Young & Bierman, 2019; =ostium for the median pharyngeal sinus sensu Dufeau & Witmer, 2015; see Ristevski et al., 2022; this foramen is the osteological correlate for the soft tissue ostium). Within the body of the parabasisphenoid are the parabasisphenoid recesses that flank the medial pharyngeal canal anterolaterally. The parabasisphenoid recesses appear as well‐defined inflations, however, they are nevertheless in communication with each other via a central recess found between them and anterior to the median pharyngeal canal. This central inflation expands dorsally within the parabasisphenoid body and gives rise to a pair of additional inflations located posteroventral to the cerebral carotid vasculature canals.

In addition to the aforementioned pneumatic cavities, the body of the parabasisphenoid is further pneumatized by anterior continuations of the median pharyngeal recess. These continuations are the precarotid, postcarotid, and subcarotid recesses (Figures 12d and 13b,d). It is unknown if the parabasisphenoid rostrum (=cultriform process) of *T. rackhami* was invaded by a rostral pneumatic recess since that portion of the parabasisphenoid is not preserved in QMF16856. The precarotid pneumatic recess is located within the anterior portion of the parabasisphenoid body, ventral to the hypophyseal fossa and cerebral carotid vascular canals. The precarotid pneumatic recess is unpaired. A relatively small (yet conspicuous) asymmetrical pair of sub‐ovoid recesses—the postcarotid recesses—occupies the anterodorsal section of the parabasisphenoid, internal to the dorsum sellae and dorsal to the hypophyseal fossa. The postcarotid recesses are laterally flanked by the abducens canals. Finally, the paired subcarotid recesses are conspicuous posterodorsally extending recesses, with the right subcarotid recess being better defined than the left. This is due to the poor preservation of the parabasisphenoid that denied precise reconstruction for some recesses.

The pharyngotympanic (middle ear) recesses, or cavities (Figures 12d and 13b,d) are significant pneumatic spaces that hollow prominent depressions (pharyngotympanic fossae; Ristevski et al., 2022) on the lateral surfaces of the prootics and medial surfaces of the quadrates. The pharyngotympanic cavities establish broad communication with the recesses within the basioccipital via the rhomboidal recesses. Two descending canals—the pharyngotympanic canals, or tubes (Figures 12d and 13b,d)—pass between the parabasisphenoid and basioccipital and exit the basicranium through the pharyngotympanic foramina (sensu Young & Bierman, 2019). Each pharyngotympanic canal has a dorsoventral length of ~4–5 mm and a diameter of ~2 mm. Due to the shortness of the median pharyngeal canal, the pharyngotympanic canals terminate on the same (the right canal) or on a slightly more ventral level (the left) than the aforementioned (Figure 12d).

Hosted within the basioccipital are large pneumatic cavities (Figures 12d and 13b,d). The occipital condyle neck and occipital condyle are the only portions of the basioccipital unaffected by the pneumatization. The basioccipital pneumatic recesses are relatively symmetrical and large. Internally, the basioccipital recesses are separated from each other by a thin (~0.3 mm) bony septum for a length of 2.5 to 3 mm. After that, the septum begins to recede within the basioccipital and so the basioccipital recesses establish communication with a large recess within the bone, a central inflation found ventral to the paired recesses. This posteroventral recess within the basioccipital expands for a length of ~3 mm before it becomes anteriorly confluent with the median pharyngeal canal.

The recessus epitubaricus (Figure 13b,d) is a ventral pneumatic extension of the pharyngotympanic cavity. Both the left and right recessus epitubaricus are in communication with the pharyngotympanic cavity posterodorsally, and the parabasisphenoid recess medially. This pneumatic recess is also in communication with the laterosphenoid pneumatic recess (read below). The cerebral carotid vascular canal separates the recessus epitubaricus from the rhomboidal recess.

Extending ventrally from each recessus epitubaricus is a pterygoid recess (Figures 12d and 13b,d). The pterygoid recesses are evident as vertical tubes that excavate the body of the parabasisphenoid and the ascending process of the pterygoid. The pterygoid recesses exit the ascending process of the pterygoid at the bone's ventral preserved portions via a pair of sub‐circular foramina (~1.5–2 mm in diameter). Whether the pterygoid recesses terminated at this point or continued to expand further within the pterygoid is unknown as the pterygoid of QMF16856 is incomplete.

Pneumatic recesses also pneumatize the laterosphenoids (Figures 12b,d and 13b,d and Figure S1.6). A complete reconstruction was achieved for the right laterosphenoid pneumatic recess, whereas the left is only partially reconstructed since the left laterosphenoid of QMF16856 is incomplete. Nevertheless, the reconstructed portion of the left laterosphenoid recess has an almost identical shape to its right counterpart, attesting to the symmetry of these recesses within each laterosphenoid. The relatively voluminous laterosphenoid recess of *T. rackhami* invades the body of the laterosphenoid. The laterosphenoid recess is somewhat constricted posteroventrally at the ventral part of the laterosphenoid, where it exits through a foramen at the laterosphenoid‐parabasisphenoid suture. Ventrally, the right laterosphenoid recess establishes communication with the recessus epitubaricus. Due to the preservational condition of the specimen, the communication between the laterosphenoid recess and the recessus epitubaricus on the left side could not be reconstructed reliably.

Within each prootic is a pneumatic cavity of the prootic facial recess (Figures 12b and 13d and Figure S1.7). The prootic facial recess occupies the element anterodorsally, at the anterior superior process of the prootic and dorsal to the trigeminal foramen. Posteriorly, each of these recesses communicates with the pharyngotympanic cavity via two sub‐oval foramina that open posteriorly to the anterior superior process of the prootic. Anterior to the prootic facial recess is the laterosphenoid recess, with which it has no direct communication. Passing immediately lateral to each prootic facial recess is the canal for the tympanic branch of the trigeminal nerve as well as the infundibular recess (with which there is no direct communication either), whereas posterior to the prootic facial recess is the endosseous labyrinth.

The intertympanic recess is a large and transversely inflated cavity (Figures 12b,d and 13b,d). This recess hollows much of the supraoccipital and the dorsal portions of the prootics. The intertympanic pneumatic recess is laterally confluent with the pharyngotympanic cavity, posteriorly with the otoccipital pneumatic recesses, and anteriorly with the parietal pneumatic recess. Anteriorly, the intertympanic recess exits the supraoccipital through two large foramina. Here, the intertympanic recess gives rise to the parietal recess (Figures 12b and 13b,d and Figure S1.5). The parietal pneumatic recess occupies much of the posterior half of the parietal bone and is located dorsal to the midbrain/hindbrain junction of the brain endocast. Although the parietal recess is unpaired, it develops five blind and asymmetrical sub‐recesses, one anteromedial and four lateral (two on each side). The external occipital vein canals pass posteromedial to the parietal recess, although the venous canal pair does not communicate with the paratympanic pneumatic system.

As suggested by their names, the otoccipital recesses occupy the otoccipital bones (Figures 12b,d and 13b) and surround the posterior portion of the brain endocast. The otoccipital pneumatic spaces are confluent with several recesses, including the intertympanic dorsally, pharyngotympanic cavities anterolaterally, and the rhomboidal recesses ventrally.

The quadrates are pneumatized by the infundibular and quadrate recesses (Figures 12b,d and 13b,d and Figure S1.8). The infundibular recess occupies the anterior half of the quadrate and is especially inflated at its anterior section. This prominently inflated anterior section of the infundibular recess is found immediately lateral to the prootic facial recess (with which it has no direct communication). Anterodorsally, each infundibular recess gives rise to an accessory inflation referred as the preinfundibular sub‐recess (see Dufeau & Witmer, 2015). Aside from being confluent with its parent recess, the preinfundibular sub‐recess has no direct communication with another component of the paratympanic pneumatic system. The preinfundibular sub‐recess is located at the anterodorsal section of the quadrate, specifically, within the anteromedial process of the element. Dorsolaterally, the infundibular recess opens to the subtympanic foramen of the quadrate. A postinfundibular recess is hardly distinguishable, although it is present as a less pronounced secondary expansion of the infundibular recess that is located ventromedially to its parent recess and is largely confluent with the pharyngotympanic cavity. Stretching posteriorly within the quadrate, the infundibular recess narrows for a relatively brief length of ~2.5 mm before it begins to expand again as it becomes continuous with the quadrate recess. The quadrate recess is housed within the body of the quadrate and is highly inflated anteriorly. The anterior inflated portion of the quadrate recess may be subdivided into two pronounced accessory recesses that are herein termed the anterolateral and anteromedial sub‐recesses of the quadrate recess. The anterolateral sub‐recess is the more voluminous of the two and is anteriorly confluent with the infundibular recess. The anteromedial sub‐recess is comparatively smaller and is in direct communication with the pharyngotympanic cavity. Between these two accessory recesses, the central inflation of the quadrate recess extends in a posterior direction as the narrow (~1.5 mm) and elongated (~9 mm) siphonium that exits the quadrate through the small foramen aëreum. The right siphoneal canal could not be reconstructed for its entire length.

## 4 DISCUSSION

### 4.1 Morphological comparisons and remarks on the neuroanatomical diversity in crocodylomorphs

#### 4.1.1 Brain endocast

Generally, the brain endocast of *T. rackhami* follows a pattern that is common among crocodylomorphs. The endocast of *T. rackhami* is relatively linear and elongated, although not to the extreme seen in the basal crocodylomorph *Almadasuchus figarii* Pol et al., 2013, the basal crocodyliform *Eopneumatosuchus colberti* Crompton & Smith, 1980, thalattosuchians, or the dyrosaurid tethysuchian *Rhabdognathus aslerensis* Jouve, 2007, where the divisions on their endocasts are arranged in a somewhat more linear fashion than most other crocodylomorphs (Brusatte, Muir, et al., 2016; Erb & Turner, 2021; Leardi et al., 2020; Melstrom et al., 2021; Pierce et al., 2017; Wilberg et al., 2021). The hypophyseal fossa of *T. rackhami*, although incomplete, is clearly inclined anterodorsally and not sub‐horizontal like that of some thalattosuchians (though note that *Macrospondylus bollensis* (von Jäger, 1828) has an inclined fossa; Herrera et al., 2018; Wilberg et al., 2021). In *Alligator mississippiensis* (Daudin, 1802), Hu et al. (2021) determined that the olfactory apparatus experiences elongation throughout life, so that larger and older alligators have a proportionally longer olfactory apparatus compared to earlier stages in ontogeny. This is supported by the measurements taken in this study, where in hatchling and juvenile *A. mississippiensis* specimens the olfactory apparatus length versus brain endocast length ratio is 0.44 and 0.42, respectively, whereas the largest specimen in the sample has a ratio of 0.58 (see Supplemental Document S4). For most taxa in the comparative sample, the olfactory apparatus length/brain endocast length ratios range from 0.4 to 0.52, and vary intraspecifically due to ontogeny, but also interspecifically. In the *T. rackhami* holotype, this ratio is 0.39. The highest ratios were estimated for the thalattosuchian *M. bollensis* and the dyrosaurid *Rh. aslerensis* (0.61 each), whereas the lowest estimated ratios belong to the notosuchian *Simosuchus clarki* Buckley et al., 2000 (0.32) and the crocodyliform *E. colberti* (0.3).

During ontogeny, the brain endocast of extant crocodylians becomes progressively elongated and with a relatively more linear organization of its divisions. In addition, the brain occupies less endocranial space in mature crocodylians as opposed to hatchlings and juveniles. The greater volume of brain versus brain endocast ratio in morphologically immature crocodylians tends to contribute toward a more compact‐looking endocast with certain brain components, like the optic lobes, having stronger impressions on the endocast. In morphologically immature individuals of extant taxa (e.g., *A. mississippiensis, C. porosus, Osteolaemus tetraspis* Cope, 1861), the midbrain region of the brain endocast is characterized by notable swellings caused by the optic lobes and the dural envelope that surrounds them (pers. obs. of a morphologically immature *O. tetraspis*, FMNH 98396; for *C. porosus*, compare Figure S2.3 with Figures S3.4 and S3.5; for *A. mississippiensis*, see Dufeau & Witmer, 2015; Hu et al., 2021). The swellings of the midbrain region induced by the optic lobes subside during ontogeny to the point where they become indistinguishable on the endocasts of mature crocodylians. The brain endocast of the *T. rackhami* holotype lacks swellings caused by the optic lobes and their adjacent dural envelope, rendering a relatively indistinct midbrain region (Figures 3, 4, 5, 6 and Figure S1.1). Thus, the brain endocast of the *T. rackhami* holotype displays morphologically mature features.

Arguably, a hallmark feature of the *T. rackhami* brain endocast is the acute dural peak over its metencephalic region. The peculiarity of this dural peak is most apparent when the endocast is observed in lateral view (Figures 5 and 6b, Figure S1.1C and S1.1D). From a lateral aspect, the sub‐triangular acute dural peak, in conjunction with the deep dorsal concavity over the midbrain region illustrate a conspicuous morphology for *T. rackhami* among hitherto described crocodylomorphs. Dural expansions over the hindbrain region of a brain endocast are not uncommon in crocodylomorphs, although their appearance in lateral aspect is variable (see the “Anatomical terminology” subsection above for definitions on the acute dural peak and blunt dural peak morphotypes). Morphologically mature brain endocasts of most crocodylian species either lack or have, at most, incipient dural inflations over the hindbrain regions. From the comparative sample available for this study (and illustrated in Figure 14 and Figure S3.1), the only eusuchians with clearly defined dural peaks are the allodaposuchids *Agaresuchus fontisensis* Narváez et al., 2016, *Ag. subjuniperus* (Puértolas‐Pascual et al., 2014) and *Arenysuchus gascabadiolorum* Puértolas‐Pascual et al., 2011, and the mekosuchine *Trilophosuchus rackhami*.

**FIGURE 15:**
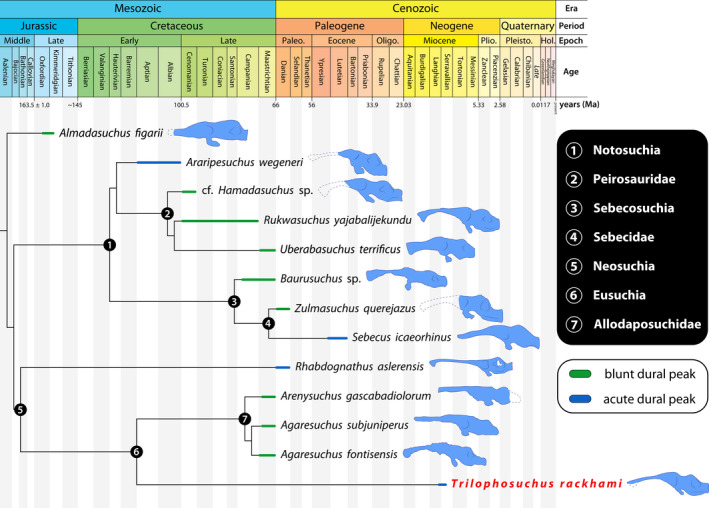
Time‐calibrated cladogram showing the relationships of crocodylomorphs that are known to possess a dorsal dural peak (as recognized in this study). Dashed lines indicate hypothetical reconstructions of missing portions of the brain endocasts. Note that *Sebecus icaeorhinus* Simpson, 1937 is tentatively accepted as possessing an acute dural peak; a digital brain endocast should clarify whether the dorsal dural peak of *Sebecus icaeorhinus* is of an acute or a blunt morphotype. The phylogenetic relationships of the notosuchians included herein are after Nicholl et al. (2021), whereas the relationships of the eusuchians are after Ristevski et al. (2022). The first and last appearance dates for the taxa were acquired from the Paleobiology Database (https://paleobiodb.org) on the 16th of June 2022. The geologic dates are based on version 2021/10 of the International Chronostratigraphic Chart (https://stratigraphy.org/chart). Brain endocast illustration of *Zulmasuchus querejazus* (Buffetaut & Marshall, 1991) after fig. 4d in Pochat‐Cottilloux et al. (2021); illustrations of *Arenysuchus gascabadiolorum* Puértolas‐Pascual et al., 2011 and *Agaresuchus subjuniperus* (Puértolas‐Pascual et al., 2014) after Figures 3k and 6f, respectively, in Puértolas‐Pascual et al. (2022). For additional illustrations of crocodylomorph brain endocasts and their sources see Figure 14 of the main text and Figure S3.1 in supplemental document S3. Abbreviations: Hol., Holocene; Oligo., Oligocene; Paleo., Paleocene; Pleisto., Pleistocene; Plio., Pliocene.

Following the established definitions, a blunt dural peak is herein recognized in several taxa: the basal crocodylomorph *Al. figarii*, the baurusuchid *Baurusuchus* sp., all peirosaurid notosuchians with currently described and/or figured endocasts (cf. *Hamadasuchus* sp., *Rukwasuchus yajabalijekundu*, and *Uberabasuchus terrificus* Carvalho et al., 2004), and (likely) the allodaposuchid eusuchians *Agaresuchus fontisensis*, *Ag. subjuniperus* and *Arenysuchus gascabadiolorum* (Figures 14a,g and 15, and Figure S3.1; see also figs. 11b in Leardi et al., 2020, 2a in George & Holliday, 2013, 6c in Sertich & O'Connor, 2014, 10c in Fonseca et al., 2020, 2c and 2d in Serrano‐Martínez et al., 2021, and 3k, 3l, 6f and 6g in Puértolas‐Pascual et al., 2022). A crocodylomorph with a likely variant of a blunt dural peak is the sebecid notosuchian *Zulmasuchus querejazus* (Buffetaut & Marshall, 1991) (see fig. 4d in Pochat‐Cottilloux et al., 2021). A noteworthy feature of *Z. querejazus* is that immediately anterior to the blunt dural peak, the dorsal surface of the brain endocast is concave. However, this concavity of *Z. querejazus* is contiguous with the blunt dural peak and is separate from a concavity over the midbrain region of the endocast (with the latter being incipient in *Z. querejazus*). While evidently not unique to peirosaurids, it is worth noting that all Peirosauridae with known brain endocasts possesses a blunt dural peak. Some variation is noticeable among these peirosaurids. For example, the endocast of *Ru. yajabalijekundu* has a deep concavity over the midbrain region. This is similar to the brain endocast of cf. *Hamadasuchus* sp. ROM 54511 (pers. obs. of a digital model, courtesy of David Dufeau), but not cf. *Hamadasuchus* sp. ROM 52620 (see fig. 2a in George & Holliday, 2013) which lacks a concavity over the midbrain region yet still has a blunt dural peak. In this regard, the endocast of cf. *Hamadasuchus* sp. ROM 52620 (as depicted in fig. 2a of George & Holliday, 2013) has similarities with that of *Z. querejazus* (the similarities are further emphasized by the comparable pontine flexures of their endocasts). Whether blunt dural peaks are ubiquitous among peirosaurids remains to be seen as the brain endocasts of other members of that clade are described in the future. Blunt dural peaks also occur in some allodaposuchid eusuchians, and they are similar in morphology to those of peirosaurids. The brain endocasts of several *Baurusuchus* sp. specimens described by Dumont Jr et al. (2020) have blunt dural peaks akin to those of peirosaurids and allodaposuchids.

Besides *T. rackhami*, an acute dural peak is also evident in the endocast reconstructions of the uruguaysuchid notosuchian *Araripesuchus wegeneri* Buffetaut, 1981 and the dyrosaurid tethysuchian *Rh. aslerensis* (Figures 14d,e and 15, and S3.1; see also figs. 22a in Sereno & Larsson, 2009 and 2b in Erb & Turner, 2021). According to the brain endocast drawing for *Sebecus icaeorhinus* Simpson, 1937 (Plate 14A in Colbert, 1946a), it seems that the prominent dural inflation in this sebecid is also with a sub‐triangular profile and as such could be regarded as an acute dural peak (Figures 14h and 15 and Figure S3.1). Therefore, based on the drawing in Colbert (1946a), I provisionally interpret the brain endocast of *S. icaeorhinus* as having an acute dural peak instead of a blunt dural peak, although note that this should be subject of reevaluation pending a description of a high‐resolution digital endocast.

The acute dural peak of *T. rackhami* is most alike to that of *Ar. wegeneri* (as reconstructed by Sereno & Larsson, 2009), where the apex of the peak terminates approximately on the same level as the dorsal‐most point of the forebrain. In contrast to *T. rackhami* and *Ar. wegeneri*, the acute dural peak of *Rh. aslerensis* terminates noticeably dorsal to the dorsal‐most point of the forebrain region (see Erb & Turner, 2021, and fig. 2a in George & Holliday, 2013). Additionally, the brain endocast of *Rh. aslerensis* is devoid of a dorsal concavity between the forebrain and hindbrain regions, whereas *T. rackhami* and *Ar. wegeneri* do have a dorsal midbrain concavity. Colbert's (1946a) illustration of the endocast of *S. icaeorhinus* depicts its dural peak terminating on a level slightly dorsal to the forebrain, although not as accentuated as in *Rh. aslerensis*. A dorsal concavity over the midbrain region may also exist in *S. icaeorhinus*, but if present, it seems to be not as deep as those in *T. rackhami* or *Ar. wegeneri*.

The condition in thalattosuchian crocodyliforms is peculiar and merits commenting. The hindbrain region of the brain endocast in thalattosuchians is characterized by a pair of hypertrophied dural venous sinus inflations that, at least superficially, may resemble one of the dural peak morphotypes defined in this study (Brusatte, Muir, et al., 2016; Dufeau, 2011; Herrera et al., 2018; Pierce et al., 2017; Schwab, Young, Herrera, et al., 2021; Wilberg et al., 2021). The dural peak morphotypes proposed here are unambiguously part of the brain endocast, such that a reconstruction of the endocast without its dural peak (where present) would be incomplete and inaccurate. Additionally, the dural peaks as defined in this study are unpaired and can be assessed independently of the external occipital vein canals (like in *T. rackhami*, for example). In thalattosuchians, the hypertrophied dural inflations are clearly paired and often extend beyond the boundaries of what is the conservatively demarcated endocast, so that they are reconstructed/marked as additional components of the same (as done by Brusatte, Muir, et al., 2016; Dufeau, 2011; Herrera et al., 2018; Schwab, Young, Herrera, et al., 2021; Wilberg et al., 2021). For the crocodyliform *E. colberti*, Melstrom et al. (2021) reported a pair of enlarged dural venous sinus inflations that are similar to those of thalattosuchians. Due to the uniqueness of the thalattosuchian condition, I consider the paired dural venous sinus inflations in thalattosuchians (as well as *E. colberti*) as distinct from the acute or blunt dural peak morphotypes defined in this study.

As described above, there are no discernable impressions of the floccular lobes on the brain endocast of *T. rackhami*. This is akin to other extant crocodylomorphs, where non‐hatchling individuals lack floccular fossae on their brain endocasts. This feature, however, is subject to ontogenetic change as according to Kuzmin et al. (2021) hatchlings of extant crocodylians do have relatively more pronounced floccular fossae. von Baczko et al. (2015) stated that absence of floccular impressions on brain endocasts is typical for extant crocodylomorphs and thalattosuchians. Nevertheless, floccular fossae are present in morphologically mature specimens of some crocodylomorphs. Thus far, floccular fossae on the dural envelope of brain endocasts have been reported in the notosuchians *Ru. yajabalijekundu* and *Si. clarki*, where they are present as relatively small and blunt lateral projections (Kley et al., 2010; Sertich & O'Connor, 2014). Floccular fossae (floccular recesses in Leardi et al., 2020) were also described on the endocast of the basal crocodylomorph *Al. figarii* (see Leardi et al., 2020). Young et al. (2021) noted that floccular fossae tend to occur in crocodylomorphs with inferred terrestrial habits (like basal crocodylomorphs and the above‐mentioned notosuchians) but are absent in semi‐aquatic and pelagic taxa (see character 25 in Young et al., 2021). However, the brain endocasts of two baurusuchid notosuchians—*Baurusuchus* sp. and *Campinasuchus dinizi* Carvalho et al., 2011—seem to lack floccular fossae (Dumont Jr et al., 2020; Fonseca et al., 2020), even though baurusuchids are considered to have been terrestrial predators (Carvalho et al., 2011; Godoy et al., 2016; Montefeltro et al., 2020; Riff & Kellner, 2011). Dumont Jr et al. (2020) suggested that the lack of floccular fossae in *Baurusuchus* sp. could be a result of small or absent cerebellar auricles and/or low ratios of brain volume to endocast volume. This discrepancy between the presence or absence of floccular fossae could indicate that impressions of the cerebellar auricles on endocasts may not be a universal feature among terrestrial crocodylomorphs. Admittedly, the relatively small sample of taxa for which there is currently data on their brain endocast morphology prevents from drawing a reliable conclusion on this matter. As such, the ontogenetic changes, intraspecific variation, and distribution of floccular fossae among adult crocodylomorphs should be studied further as more data becomes available.

#### 4.1.2 Endosseous labyrinths

**FIGURE 16:**
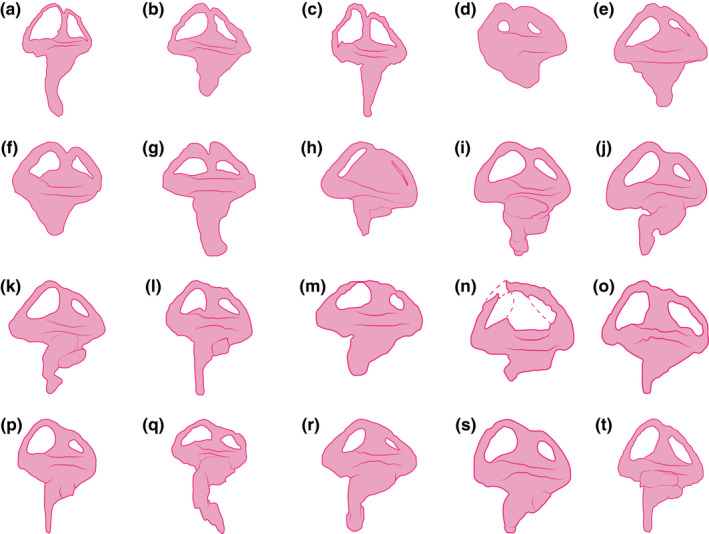
Endosseous labyrinth silhouettes of select crocodylomorphs, depicted in lateral view. Dashed lines indicate hypothetical reconstructions of missing portions. Illustrations not to scale. Endosseous labyrinth illustration of (a) *Junggarsuchus sloani* Clark et al., 2004, (b) *Eopneumatosuchus colberti* Crompton & Smith, 1980, (c) *Protosuchus haughtoni* (Busbey III & Gow, 1984), (d) *Cricosaurus araucanensis* (Gasparini & Dellapé, 1976), (e) *Macrospondylus bollensis* (von Jäger, 1828), (f) ‘*Metriorhynchus*’ cf. *M*. *brachyrhynchus*, (g) *Plagiophthalmosuchus* cf. *P*. *gracilirostris*, (h) *Rhabdognathus aslerensis* Jouve, 2007, (i) *Alligator mississippiensis* (Daudin, 1802), (j) *Caiman crocodilus* (Linnaeus, 1758), (k) *Crocodylus johnstoni* (Krefft, 1873) (l) *Crocodylus rhombifer* Cuvier, 1807, (m) *Gavialis gangeticus* (Gmelin, 1789), (n) *Gryposuchus neogaeus* (Burmeister, 1885), (o) *Gunggamarandu maunala* Ristevski et al., 2021, (p) *Mecistops cataphractus* (Cuvier, 1824), (q) *Mourasuchus arendsi* Bocquentin Villanueva, 1984, (r) *Osteolaemus tetraspis* Cope, 1861, (s) *Tomistoma schlegelii* (Müller, 1838), and (t) *Trilophosuchus rackhami* Willis, 1993. For an expanded version of this figure see Figure S3.2 in supplemental document S3. The sources used to create these illustrations are given in Table S2.3 of supplemental document S2.

In lateral and medial views, the vestibular apparatus of *T. rackhami* is sub‐pyramidal (Figures 10a,b and 16t, Figure S1.3A, S1.3B, S1.4A, and S1.4B). The markedly pyramidal vestibular apparatus of *T. rackhami*, particularly its dorsal contour (as formed by the anterior and posterior semicircular canals dorsal to the common crus), is noticeably different from crocodylians which have more smoothly rounded anterior and posterior semicircular canals upon their dorsal contact with the common crus (e.g., *Crocodylus acutus* Cuvier, 1807, *C. intermedius* Graves, 1819, *C. porosus*, *Gavialis gangeticus* (Gmelin, 1789), *Gryposuchus neogaeus* (Burmeister, 1885), *Mourasuchus arendsi* Bocquentin Villanueva, 1984, *Tomistoma schlegelii* (Müller, 1838); Figure 16m,n,q,s and Figure S3.2; pers. obs. of QMJ48127 and QMJ52809; figs 5e in Bona et al., 2013, 8c–8f in Brusatte, Muir, et al., 2016, 8 in Bona et al., 2017, and 4.1.8D in Serrano‐Martínez, 2018). To a lesser degree, the endosseous labyrinth of *T. rackhami* somewhat resembles the more accentuated sub‐pyramidal profiles of the labyrinths of *Junggarsuchus sloani* Clark et al., 2004 and *Protosuchus haughtoni* (Busbey III & Gow, 1984) (Figure 16a,c and Figure S3.2). However, in this context, it is important to state that the semicircular canals of *T. rackhami* are not as slender as those of some basal crocodylomorphs and crocodyliforms, or certain sebecosuchian notosuchians (e.g., *Z. querejazus*; see Pochat‐Cottilloux et al., 2021) and are more alike to those of other eusuchians (see Schwab et al., 2020). Also, the common crus of *T. rackhami* is relatively robust, by being tapered dorsally yet wide at the base, and thus differing from basal crocodylomorphs like *J. sloani* which has a slender common crus. In most crocodylomorphs, the ratio of the endosseous cochlear duct height compared to the total height of the endosseous labyrinth is ~0.5. *Trilophosuchus rackhami* also falls within this bracket by having an endosseous cochlear duct height/total endosseous labyrinth height ratio of 0.5.

One of the most peculiar features of the labyrinth of *T. rackhami* concerns the height ratio of the common crus when compared to the total height and anteroposterior length of the endosseous labyrinth, and the ratio of the height of the common crus compared to the height of the vestibular apparatus (Table 2). *Trilophosuchus rackhami* has one of the highest ratios among the sampled crocodylomorphs. The highest ratio of common crus height/endosseous labyrinth height is estimated for an undescribed “sphenosuchian” crocodylomorph (0.29), followed by *T. rackhami* (0.23), then the thalattosuchians *Cricosaurus araucanensis* (Gasparini & Dellapé, 1976) and *Pelagosaurus typus* Bronn, 1841 (0.21 each), and the baurusuchid *Baurusuchus* sp. (0.2). All remaining taxa have common crus height/endosseous labyrinth height ratios lower than 0.2, with the alligatorid *Mo. arendsi* having the lowest estimate (0.09). Since the endosseous cochlear duct is not always reconstructed for its full length (usually missing the ventral‐most, or lagenar, portion), the endosseous labyrinth height ratio could not be accurately estimated for most crocodylomorphs in the comparative sample. Also, the reconstructed length of the endosseous cochlear duct in some metriorhynchid thalattosuchians, such as *Cri. araucanensis*, is proportionately short which in turn skews the results of a proportionally tall common crus. Schwab et al. (2020) and Schwab, Young, Herrera, et al. (2021) remarked that short endosseous cochlear ducts could potentially be a feature of Metriorhynchidae, although they did not exclude the possibility that the reconstructed lengths could be short because of a segmentation bias. Fortunately, the ratio of the common crus height/endosseous labyrinth anteroposterior length and common crus height/vestibular apparatus height could be estimated for a greater number of taxa. Even then, *T. rackhami* has a ratio that ranks among the five highest. For the common crus height/endosseous labyrinth anteroposterior length, the undescribed “sphenosuchian” is the one with the highest ratio (0.36), followed by the basal crocodylomorph *J. sloani* (0.33), *Baurusuchus* sp. (0.31), *T. rackhami* (0.3), and in fifth place are the basal crocodyliform *Pr. haughtoni* and the sebecid *Z. querejazus* (0.29 each). All remaining taxa have ratios lower than 0.29, with the lowest estimate in this category made for the thalattosuchian *Plagiophthalmosuchus* cf. *Pl. gracilirostris* (0.12). In the third category (common crus height/vestibular apparatus height), *T. rackhami* is once again ranking among the top five. Again, the undescribed “sphenosuchian” has the highest ratio (0.44), followed by *J. sloani* (0.42), *Baurusuchus* sp. (0.4), and tied in fourth place are *T. rackhami* along with *E. colberti* and *Pe. typus* (0.38 each). In fifth place are the extant *C. niloticus* Laurenti, 1768 (0.36) and *Gav. gangeticus*. The remaining taxa have ratios of 0.33 or less, with the lowest ratio in this category estimated for the extant *Caiman crocodilus* (Linnaeus, 1758) (0.13). Out of the five *Gav. gangeticus* specimens that were measured, only one has a ratio of 0.36 for the common crus height/vestibular apparatus height category, while the other four have lower ratios that range from 0.25 to 0.29. Indeed, taxa measured from multiple specimens (mostly extant) result in variable ratios, indicating that the proportions of the common crus height are subject to a certain degree of intraspecific (but also ontogenetic) variation. Regardless, it is interesting to note that *T. rackhami* consistently ranks among those with the greatest common crus height ratios, and is usually accompanied by basal crocodylomorphs, basal crocodyliforms, sebecosuchians, or few thalattosuchians. While some eusuchians do have relatively high common crus ratios, none are as great as those of *T. rackhami*. The functional implications of the tall common crus, if any, are unclear.

**TABLE 2:** Ratio of common crus height vs endosseous labyrinth height and anteroposterior length, and vestibular apparatus height in select crocodylomorphs. The ratios are based on measurements of subadult or adult individuals. Ratios that could not be estimated have omitted data (indicated with a dash). Abbreviations: CCRH, common crus height; ELH, endosseous labyrinth height; ELL, endosseous labyrinth anteroposterior length; VEH, vestibular apparatus height

Taxon	CCRH/ELH	CCRH/ELL	CCRH/VEH
*Alligator mississippiensis*	0.14–0.19	0.16–0.27	0.23–0.33
*Almadasuchus figarii*	—	0.25	—
*Baurusuchus* sp.	0.2	0.31	0.4
*Caiman crocodilus*	0.15	0.2	0.13
*Cricosaurus araucanensis*	0.21	0.22	0.33
*Cricosaurus schroederi*	—	0.15	0.21
*Crocodylus acutus*	0.14	0.18	0.25
*Crocodylus johnstoni*	0.17	0.21	0.3
*Crocodylus moreletii*	0.13–0.14	0.19–0.21	0.25
*Crocodylus niloticus*	—	0.22	0.36
*Crocodylus porosus*	0.15	0.17–0.19	0.17–0.22
*Crocodylus rhombifer*	0.15	0.19	0.23
*Crocodylus siamensis*	—	0.2	0.3
*Eopneumatosuchus colberti*	—	0.24	0.38
*Gavialis gangeticus*	0.14	0.18–0.25	0.25–0.36
*Gunggamarandu maunala*	—	0.21	0.28
cf. *Hamadasuchus* sp.	0.12–0.15	0.2–0.21	0.29
*Junggarsuchus sloani*	0.19	0.33	0.42
*Macrospondylus bollensis*	0.14–0.16	0.18–0.19	0.3–0.33
*Mecistops cataphractus*	0.17	0.24	0.31
*Melanosuchus niger*	—	0.19	0.3
*‘Metriorhynchus’* cf. *‘M.’ brachyrhynchus*	0.14	0.15	0.23
*Mourasuchus arendsi*	0.09	0.14	0.21
*Osteolaemus tetraspis*	0.18	0.19	0.25–0.33
*Paleosuchus palpebrosus*	—	0.18	0.23
*Pelagosaurus typus*	0.21	0.27	0.38
*Plagiophthalmosuchus* cf. *P. gracilirostris*	0.12	0.12	0.18
*Protosuchus haughtoni*	0.17	0.29	0.33
*Thalattosuchus superciliosus*	0.12	0.14	0.19
*Tomistoma schlegelii*	0.13–0.16	0.16–0.18	0.23–0.24
*Torvoneustes coryphaeus*	—	0.16	—
*Trilophosuchus rackhami*	0.23	0.3	0.38
Undescribed “sphenosuchian”	0.29	0.36	0.44
*Zulmasuchus querejazus*	—	0.29	0.26

#### 4.1.3 Cranial pneumaticity

The crocodylomorph braincase is invaded by a complex system of paratympanic pneumatic recesses, although the degree of cranial pneumaticity is diverse between species and even higher clades within Crocodylomorpha (e.g., Busbey III & Gow, 1984; Crompton & Smith, 1980; Dufeau, 2011; Kuzmin et al., 2021; Leardi et al., 2017, 2020; Melstrom et al., 2021; Pierce et al., 2017; Walker, 1990; Wu & Chatterjee, 1993). Many studies have surveyed the developmental patterns and morphological diversity of the paratympanic pneumaticity in extant crocodylians (e.g., Bona et al., 2017; Colbert, 1946b; Dufeau & Witmer, 2015; Dufeau, 2011; Gold et al., 2014; Iordansky, 1973; Kuzmin et al., 2021; Owen, 1850; Pierce et al., 2017; Serrano‐Martínez, 2018; Serrano‐Martínez et al., 2019a; Tarsitano, 1985; Witmer et al., 2008). The crocodylian paratympanic pneumatic system is heavily influenced by ontogenetic changes, with adults having comparatively less developed pneumatic diverticula than hatchlings and juveniles (Dufeau, 2011; Dufeau & Witmer, 2007, 2015; Kuzmin et al., 2021). This reduction in cranial pneumaticity is expressed by the diminishing volume of the pneumatic diverticula and in some cases, even the loss of certain diverticula that used to be present earlier in ontogeny. Interspecific variation is another factor in the development of pneumaticity, not only within Crocodylia but Crocodylomorpha in general (Dufeau, 2011; Kuzmin et al., 2021). At present, the palaeoneurology of most extinct crocodylomorphs, including their paratympanic pneumaticity, is understudied. The cranial pneumaticity in many extinct crocodylians from the Cenozoic of Australia is yet to be assessed, although limited data are available on the gavialoids *G. maunala* and *Ha. camfieldensis*, as well as the mekosuchine *P. vincenti* (Megirian et al., 1991; Ristevski et al., 2020a, 2020b, 2021).

The holotype of *T. rackhami* has a highly pneumatic braincase (Figures 11, 12, 13). When compared to extant taxa, the pneumatic recesses of QMF16856 are most similar to those of *O. tetraspis* (pers. obs. of FMNH 98396; see also Kuzmin et al., 2021) and *Paleosuchus palpebrosus* Cuvier 1807, (see figs. 1–7a in Dufeau, 2011). Like *O. tetraspis* and *Pa. palpebrosus*, the holotype of *T. rackhami* has a voluminous pneumatic recess within the laterosphenoid that communicates with the recessus epitubaricus, a prootic facial recess (although according to Kuzmin et al., 2021, it may be absent in some *Pa. palpebrosus* specimens), a spacious parietal recess, and a highly pneumatized basicranium (basioccipital and parabasisphenoid). Compared to the immature *O. tetraspis* FMNH 98396, the holotype of *T. rackhami* has a more expansive cranial pneumaticity, such as within the basicranium, but also the laterosphenoid pneumatic recess which is more capacious in QMF16856. As reported by Kuzmin et al. (2021), extant gavialids and crocodylids (bar osteolaemine crocodylids) have more reduced paratympanic recesses than extant alligatorids. Therefore, it appears that the general developmental degree of paratympanic pneumaticity in *T. rackhami* is more akin to extant members of Alligatoroidea than to Longirostres (Crocodyloidea + Gavialoidea)—with the exception in Longirostres being the osteolaemine crocodylids, like *O. tetraspis*.

Compared to extinct crocodylomorphs (where known), the pneumatized cranium of *T. rackhami* is most similar to those of basal crocodylomorphs and crocodyliforms, and notosuchians (Dufeau, 2011; Fonseca et al., 2020; Leardi et al., 2020; Pochat‐Cottilloux et al., 2021), although the degree of cranial pneumaticity in the *T. rackhami* holotype is not as developed. As can be interpreted from figs. 1–5 and 1–7a–c in Dufeau (2011), the crocodyliform *E. colberti* and the notosuchian cf. *Hamadasuchus* sp. have greatly pneumatized crania, more so than the *T. rackhami* holotype. The quadrates of cf. *Hamadasuchus* sp. are especially inflated (also in *Al. figarii*), which contrasts with *T. rackhami* where the quadrates, although highly pneumatic, are not as hollow as those in the aforementioned. While not unalike *T. rackhami*, the baurusuchid Ca. *dinizi* has a somewhat more expansive paratympanic pneumatic system, as does the sebecid *Z. querejazus* (Fonseca et al., 2020; Pochat‐Cottilloux et al., 2021). Otherwise, the cranium of *T. rackhami* is substantially more pneumatic than that of thalattosuchians or the dyrosaurid tethysuchian *Rh. aslerensis*. Paratympanic pneumaticity is reduced in thalattosuchians, a group that evolved highly aquatic forms, and some thalattosuchians (Metriorhynchidae) were even pelagic (Brusatte, Muir, et al., 2016; Dufeau, 2011; Fernández et al., 2011; Herrera et al., 2018; Pierce et al., 2017; Schwab, Young, Herrera, et al., 2021; Wilberg et al., 2019, 2021). A more aquatic palaeoecology than that of extant crocodylians has been hypothesized for dyrosaurid tethysuchians (Wilberg et al., 2019), such as *Rh. aslerensis* which also has relatively reduced paratympanic pneumaticity (Erb & Turner, 2021). Ultimately, the cranial pneumaticity of the *T. rackhami* holotype appears more comparable to that of certain eusuchians. However, *T. rackhami* has one of the most pneumatic crania within Eusuchia (at least in comparison to eusuchians that have information on their paratympanic pneumatic system as published to date).

Adult *O. tetraspis* and *Pa. palpebrosus* are characterized by crania that are more pneumatized than adults of some other taxa, such as *C. porosus* or *Gav. gangeticus* (Bona et al., 2017; Dufeau, 2011; Kuzmin et al., 2021; Pierce et al., 2017; pers. obs. of *C. porosus* specimens QMJ48127 and QMJ52809). In addition to their extensive cranial pneumaticity, *Pa. palpebrosus* and *O. tetraspis* also possess altirostral snouts and correspondingly deep braincases (Dufeau, 2011). Kuzmin et al. (2021) stated that despite their distant phylogenetic relationships, *Pa. palpebrosus* (Alligatoridae) and *O. tetraspis* (Crocodylidae) share comparable cranial morphologies and similar ecological preferences. Out of all extant crocodylians, the gross cranial morphology of *T. rackhami* is most alike to (but nonetheless distinct from) *O. tetraspis* and *Pa. palpebrosus*, by having a relatively deep braincase and an (inferred) altirostral snout morphology (Ristevski et al., 2022; Willis, 1993). Willis et al. (1993) considered the morphological resemblances between *T. rackhami* and the extant *O. tetraspis* and *Pa. palpebrosus* to be a result of convergence on a similar cranial adaptive morphology. The morphological likeness of the paratympanic pneumaticity between *T. rackhami* and other similarly deep‐snouted crocodylomorphs, both extant and extinct, is not surprising. According to Dufeau (2011), Dufeau & Witmer (2015) and Kuzmin et al. (2021), the morphological variety of the paratympanic pneumatic system in crocodylians is a consequence of biomechanical constraints imposed by the overall cranial morphology, as well as bones of the braincase and their adjacent soft tissue (i.e., muscles and vascular elements). Taxa with reduced cranial pneumaticity like thalattosuchians and *Rh. aslerensis* have long and narrow snouts, but also correspondingly narrow braincases in order to accommodate the enlarged jaw adductor muscles. Such morphology constraints the development of the paratympanic pneumaticity in crocodylomorphs with proportionally large jaw adductor musculature. Because *T. rackhami* has a relatively deep braincase and an inferred altirostral snout, its cranial pneumaticity is developed like that of crocodyliforms with a similar skull morphology (e.g., *O. tetraspis*, *Pa. palpebrosus*, and to a comparable yet somewhat lesser degree, Ca. *dinizi*, cf. *Hamadasuchus* sp., and *Z. querejazus*).

## 5 CONCLUSIONS

While the neuroanatomy of *Trilophosuchus rackhami* adheres to the general morphological patterns witnessed in crocodylomorphs, it is nevertheless represented by a unique combination of features that collectively stand out among currently known taxa. Compared to crocodylomorphs with known aspects of their neuroanatomy, the overall neuromorphology of *T. rackhami* is similar to the extant *Osteolaemus tetraspis* and *Paleosuchus palpebrosus*, but it also resembles some notosuchians. The gross morphology of the brain endocast of *T. rackhami* appears similar, but not identical, to that of the notosuchian *Araripesuchus wegeneri*. The similarities between these two taxa are highlighted by the comparable lateral outlines of their acute dural peaks over the hindbrain regions of the brain endocasts. The notosuchian *Sebecus icaeorhinus* also seems to possess an acute dural peak that, while distinct, appears most alike to those of *T. rackhami* and *Ar. wegeneri*. A dural peak over the dorsal surface of a brain endocast is a feature present in several crocodylomorphs, with two dural peak morphotypes being recognized in this study—an acute and a blunt dural peak. In *T. rackhami*, the common crus of the endosseous labyrinth is unusually tall, and has one of the greatest height ratios among the sampled taxa. The paratympanic pneumatic system of the *T. rackhami* holotype is greatly developed. Although resemblances are evident in the developmental degree between the paratympanic pneumaticity of *T. rackhami* and some basal crocodylomorphs, basal crocodyliforms and notosuchians, ultimately, *T. rackhami* has a paratympanic pneumatic system that is more similar to those of eusuchians. Relative to extant crocodylians, the cranial pneumaticity of the *T. rackhami* holotype is similar yet somewhat greater than in taxa with the most pneumatic crania, such as *O. tetraspis* and *Pa. palpebrosus*. Some morphological aspects of the brain endocast (dural peak morphotypes), endosseous labyrinths (common crus height ratios), and paratympanic pneumaticity discussed in this study may have phylogenetic potential that merit exploration in future systematic studies.

## Data Availability

All supplementary data is available at https://doi.org/10.5061/dryad.fbg79cnx4 and https://doi.org/10.5281/zenodo.6968373. The supplementary material includes interactive 3D PDF files, 3D digital models, measurements, and additional information on crocodylomorph neuroanatomy.
